# Supplementary material for: Diverse immune response of DNA damage repair-deficient tumors
Source: Cell Rep Med. 2021 May 18;2(5):100276. doi: 10.1016/j.xcrm.2021.100276 (PMC8149377; doi:10.1016/j.xcrm.2021.100276)
Supplement: Document S1. Figures S1–S5 and Tables S1 and S2 [file mmc1.pdf]

Cell Reports Medicine, Volume *2*

Supplemental information

Diverse immune response of DNA damage repair-deficient tumors

Tao Qing, Tomi Jun, Katherine E. Lindblad, Amaia Lujambio, Michal Marczyk, Lajos Pusztai, and Kuan-lin Huang

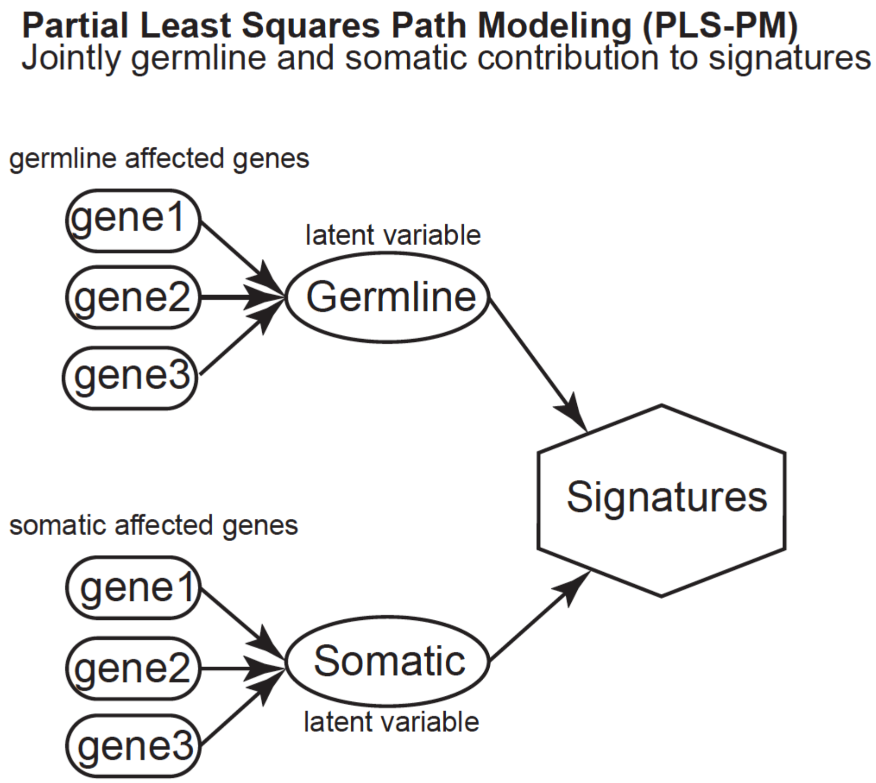

Figure S1. A schema of Partial Least Squares Path Modeling analysis, related to Figure 2E. Two latent variables are introduced, representing the combined effects of germline affected genes and somatic affected genes. The signatures indicate genome damage signatures, including tumor mutation burden, SNV, and indel neoantigen loads.

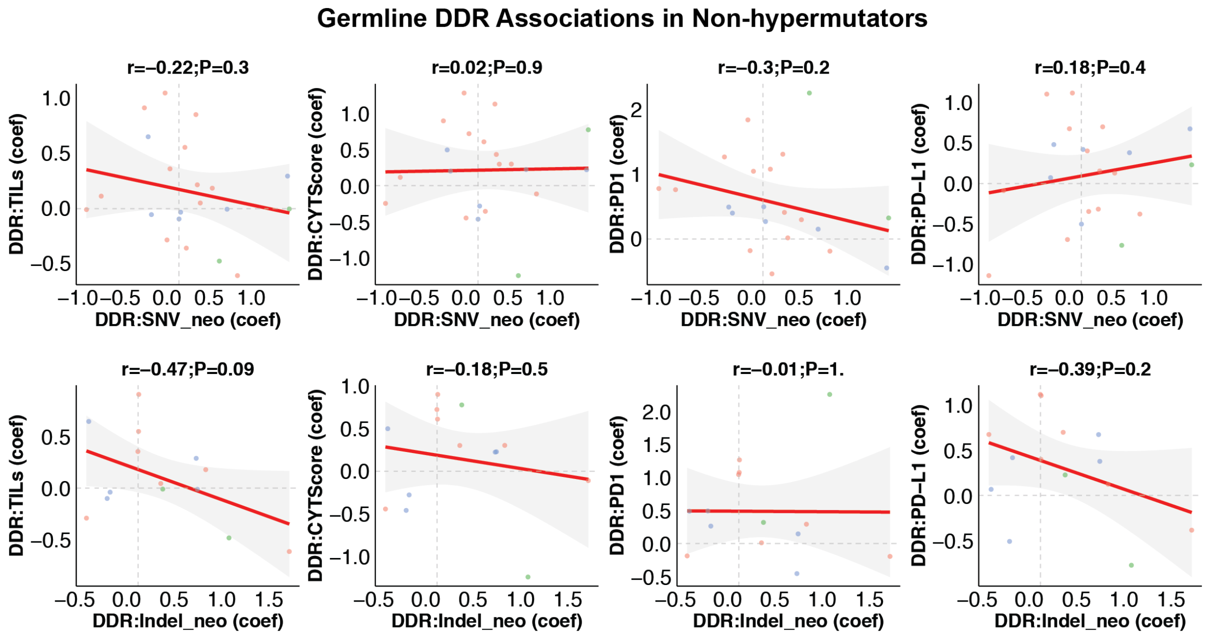

Figure S2. The association between germline DNA damage repair and tumor immune response in non-hypermutated cases, related to Figure 4. A-D. Comparisons of the correlation coefficients of germline DDR versus SNV neoantigen load with the germline DDR versus immune signature, including **A.** TILs, **B.** CYT score, **C.** *PD1* expression, and **D.** *PD-L1* expression. **E-H**. Comparisons of the correlation coefficients of germline DDR versus Indel neoantigen load with the germline DDR versus immune signature, including **E.** TILs, **F.** CYT score, **G.** *PD1* expression, and **H.** *PD-L1* expression. The *r* represents the Pearson correlation coefficient. Color of the dot represents the DDR pathways, including MMR (*MLH1*, *MSH2*, *MSH3*, *MSH6*, and *PMS2*), HR (*BRCA1/2, PALB2*), Sensor (*ATM, ATR,* and *CHEK2*) and Polymerase (*POLE* and *POLQ*). The axes indicate the correlation coefficients (coef) of linear regression adjusted by patients' age and genetic components (*immune signature/neoantigen load ~ germline DDR variants+Age+PC1+PC2*).

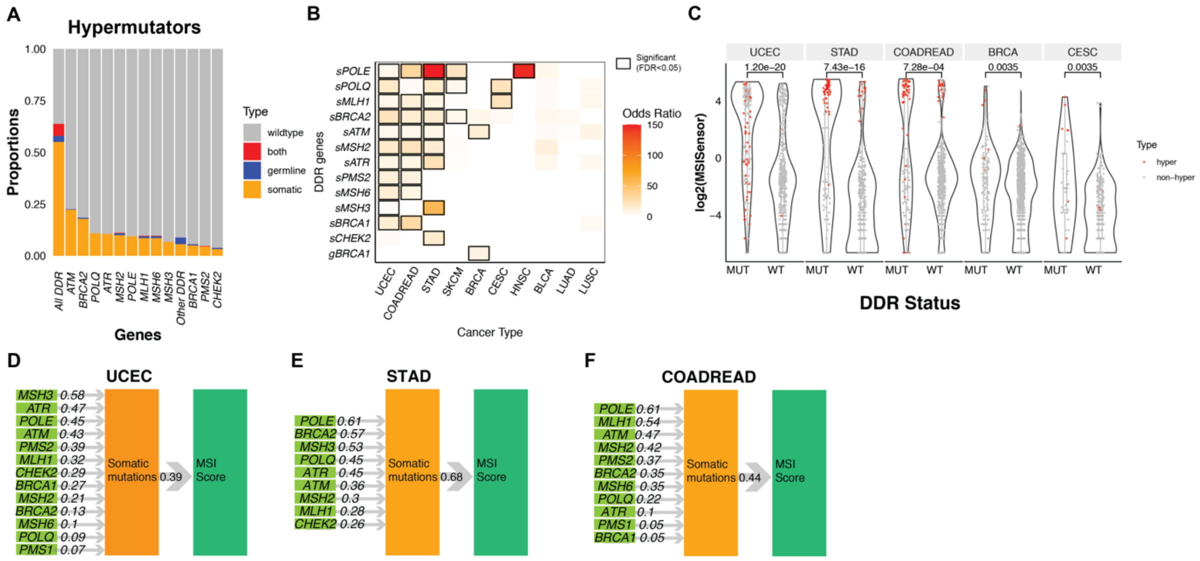

Figure S3. The effect of germline and somatic DNA damage repair mutations on hypermutator and microsatellite instability, related to Figure 2 & 3. A. The frequency of hypermutators carrying germline variants or somatic mutations in any of the 80 DDR genes. **B.** The enrichment of 13-prioritized DDR gene mutations in hypermutators, where the FDR was calculated based on results of two-side Fisher's exact tests. Only cancer types with at least one FDR<0.15 were shown. **C.** The comparison of MSISensor scores in DDR mutated and wildtype samples in each cancer type based on the Wilcox-rank-sum test. The numbers on the top of the violin plots denote FDRs resulting from the comparison. Only show cancer types with FDR<0.05 were shown. **D-F.** PLS-PM models of somatic mutations of DDR genes and MSI score (germline level was only show, because no significant association was identified in the germline level), including **D.** UCEC, **E.** STAD, **F.** COADREAD. The numbers indicated correlation coefficients identified by the PM-PLS model. Coefficients between germline variant/somatic mutation nodes and MSI nodes indicate the combined associations of the listed DDR genes.

A

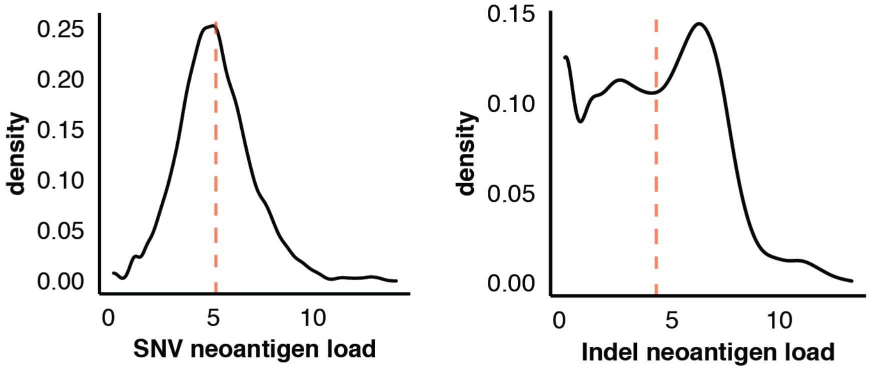

B

COADREAD

Odds Ratio=46.5 , *P*<2.2e-16

	SNV Neo High	SNV Neo Low
**MLH1-deficiency**	54 (11.2%)	4 (1.0%)
**MLH1-wildtype**	79 (16.4%)	344 (71.4%)

COADREAD

Odds Ratio=19.1 , *P*=1.46e-14

	Indel Neo High	Indel Neo Low
**MLH1-deficiency**	51 (11.5%)	4 (0.9%)
**MLH1-wildtype**	155 (35.0%)	233 (52.6%)

Figure S4. Neoantigen load and immunogenicity of *MLH1*-deficiency tumor, related to Figure 6. **A.** The distribution of log2-scale SNV and indel neoantigen load. The red line indicates the average neoantigen load for stratifying neoantigen high and low samples. **B.** contingency tables for *MLH1*-deficiency (germline variants, somatic mutations, and DNA methylation) and SNV/indel neoantigens. *P*-values were calculated by two-sided Fisher exact test.

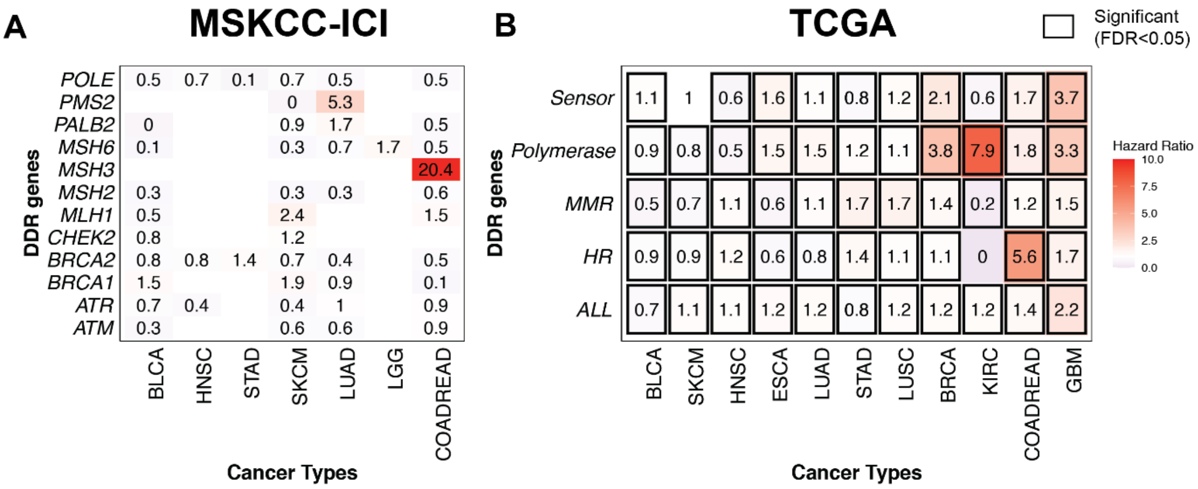

Figure S5. Associations between patients' outcome and somatic mutations of the 13-prioritized DDR genes, related to Figure 7. A. The heatmap shows the hazard ratio of individual DDR genes in the MSKCC ICI treatment cohort. The X-axis represents cancer types, and the Y-axis represents somatic mutations in each prioritized DDR gene. **B.** The heatmap shows the hazard ratio of DDR pathways in the MSKCC ICI treatment cohort. The Y-axis represents somatic mutations in DDR pathways of prioritized DDR genes, including Mismatch Repair (MMR), Homologous Recombination (HR), Damage Sensor (Sensor), DNA Polymerase (Polymerase), and all the 13-prioritized DDR genes (ALL). The value in each cell denotes the hazard ratio. The hazard ratio > 1 and < 1 suggests the association of DDR mutations with worse and better survival, respectively. Hazard ratio and *P* values were calculated using multivariable Cox proportional hazards models. Black boxes indicate FDR meet the criteria of less than 0.05 (significant).

Supplementary Table1: TCGA samples, related to Figure 1.

NO.	Cancers	Abbreviation	# All Samples	# Hypermutators
1	Glioblastoma multiforme	GBM	390	3
2	Ovarian serous cystadenocarcinoma	OV	387	1
3	Lung adenocarcinoma	LUAD	515	27
4	Lung squamous cell carcinoma	LUSC	485	16
5	Prostate adenocarcinoma	PRAD	497	2
6	Uterine Corpus Endometrial Carcinoma	UCEC	529	51
7	Bladder Urothelial Carcinoma	BLCA	411	18
8	Testicular Germ Cell Tumors	TGCT	129	0
9	Esophageal carcinoma	ESCA	184	3
10	Pancreatic adenocarcinoma	PAAD	177	1
11	Kidney renal papillary cell carcinoma	KIRP	281	0
12	Liver hepatocellular carcinoma	LIHC	363	3
13	Cervical squamous cell carcinoma and endocervical adenocarcinoma	CESC	289	14
14	Sarcoma	SARC	236	2
15	Breast invasive carcinoma	BRCA	1012	12
16	Thymoma	THYM	123	1
17	Mesothelioma	MESO	82	0
18	Colon Rectum adenocarcinoma	COADREAD	527	64
19	Stomach adenocarcinoma	STAD	439	67
20	Skin Cutaneous Melanoma	SKCM	466	44
21	Cholangiocarcinoma	CHOL	36	1
22	Kidney renal clear cell carcinoma	KIRC	368	0
23	Thyroid carcinoma	THCA	492	0
24	Head and Neck squamous cell carcinoma	HNSC	507	5
25	Brain Lower Grade Glioma	LGG	511	1
26	Kidney Chromophobe	KICH	66	1
27	Uterine Carcinosarcoma	UCS	57	2
28	Adrenocortical carcinoma	ACC	92	2
29	Pheochromocytoma and Paraganglioma	PCPG	179	0
30	Uveal Melanoma	UVM	80	0
31	Lymphoid Neoplasm Diffuse Large B-cell Lymphoma	DLBC	37	0
32	Acute Myeloid Leukemia	LAML	133	1

Supplementary Table2: core DNA damage repair genes, related to Figure 1

DNA Damage Repair Pathways	Genes	#Gene
Base Excision Repair (BER)	APEX1,APEX2,FEN1,PARP1,POLB,TDG,TDP1,UNG	8
Nucleotide Excision Repair (NER, including TC-NER and GC-NER))	CUL5,ERCC1,ERCC2,ERCC4,ERCC5,ERCC6,POLE, POLE3,XPA,XPC	10
Mismatch Repair (MMR)	EXO1,MLH1,MLH3,MSH2,MSH3,MSH6,PMS1,PMS2	8
Fanconi Anemia (FA)	FANCA,FANCB,FANCC,FANCD2,FANCI,FANCL, FANCM,UBE2T	8
Homologous Recombination (HR)	BARD1,BLM,BRCA1,BRCA2,BRIP1,EME1,GEN1,MRE11A,MUS81,NBN,PALB2,RAD50,RAD51,RAD52, RBBP8,SHFM1,SLX1A,TOP3A,TP53BP1,XRCC2, XRCC3	21
Non-homologous End Joining (NHEJ)	LIG4,NHEJ1,POLL,POLM,PRKDC,XRCC4,XRCC5, XRCC6	8
Direct Repair (DR)	ALKBH2,ALKBH3,MGMT	3
Translesion Synthesis (TLS)	POLN,POLQ,REV1,REV3L,SHPRH	5
Damage Sensor etc.	ATM,ATR,ATRIP,CHEK1,CHEK2,MDC1,RNMT, TOPBP1,TREX1	9
