# Supplementary material for: Diverse immune response of DNA damage repair-deficient tumors
Source: Cell Rep Med. 2021 May 18;2(5):100276. doi: 10.1016/j.xcrm.2021.100276 (PMC8149377; doi:10.1016/j.xcrm.2021.100276)
Supplement: Document S2. Article plus supplemental information [file mmc5.pdf]

Cell Reports
Medicine

Article

Diverse immune response of DNA damage repair-deficient tumors

Graphical abstract

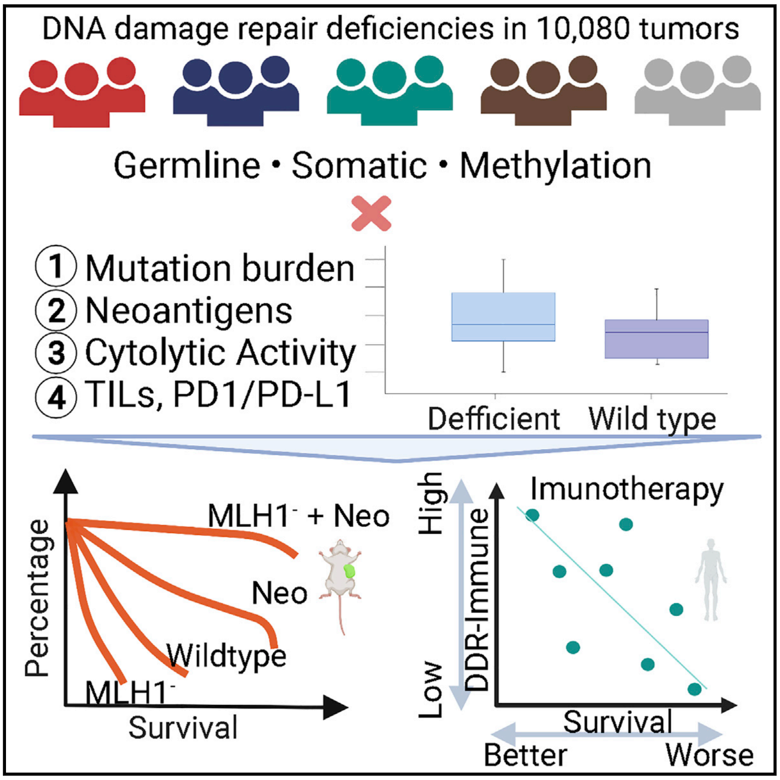

Highlights

- Tumor immunogenicity is associated with DNA damage repair deficiencies (DDR-ds)
- The immunogenicity of DDR alterations varies by pathways and cancer types
- DDR-d tumors with high immune infiltrates correlate with immunotherapy response

Authors

Tao Qing, Tomi Jun, Katherine E. Lindblad, Amaia Lujambio, Michal Marczyk, Lajos Pusztai, Kuan-lin Huang

Correspondence

lajos.pusztai@yale.edu (L.P.), kuan-lin.huang@mssm.edu (K.-l.H.)

In brief

Qing et al. systematically compare neoantigen loads, immune infiltrates, and immunotherapy responses in tumors harboring different germline, somatic, and methylation alterations of DNA damage repair genes. Immunogenicity is affected by the affected DDR pathway(s) and cancer types, and specific DDR alterations may provide more precise biomarkers for immunotherapy.

Qing et al., 2021, Cell Reports Medicine 2, 100276
May 18, 2021 © 2021 The Author(s).
https://doi.org/10.1016/j.xcrm.2021.100276

CellPress

Cell Reports Medicine

CellPress
OPEN ACCESS

Article

Diverse immune response of DNA damage repair-deficient tumors

Tao Qing,[1] Tomi Jun,[2] Katherine E. Lindblad,[3,4,5,6] Amaia Lujambio,[3,4,5] Michal Marczyk,[1,7] Lajos Pusztai,[1,*] and Kuan-lin Huang[8,9,*]

[1]Breast Medical Oncology, Yale School of Medicine, New Haven, CT 06511, USA
[2]Division of Hematology and Medical Oncology, Tisch Cancer Institute, Icahn School of Medicine at Mount Sinai, New York, NY 10029, USA
[3]Department of Oncological Sciences, Icahn School of Medicine at Mount Sinai, New York, NY 10029, USA
[4]Liver Cancer Program, Division of Liver Diseases, Department of Medicine, Tisch Cancer Institute, Icahn School of Medicine at Mount Sinai, New York, NY 10029, USA
[5]The Precision Immunology Institute, Icahn School of Medicine at Mount Sinai, New York, NY 10029, USA
[6]Graduate School of Biomedical Sciences at Icahn School of Medicine at Mount Sinai, New York, NY 10029, USA
[7]Department of Data Science and Engineering, Silesian University of Technology, Gliwice, Poland
[8]Department of Genetics and Genomic Sciences, Center for Transformative Disease Modeling, Tisch Cancer Institute, Icahn Institute for Data Science and Genomic Technology, Icahn School of Medicine at Mount Sinai, New York, NY 10029, USA
[9]Lead contact
*Correspondence: lajos.pusztai@yale.edu (L.P.), kuan-lin.huang@mssm.edu (K.-l.H.)
https://doi.org/10.1016/j.xcrm.2021.100276

SUMMARY

Tumors with DNA damage repair (DDR) deficiency accumulate genomic alterations that may serve as neoantigens and increase sensitivity to immune checkpoint inhibitor. However, over half of DDR-deficient tumors are refractory to immunotherapy, and it remains unclear which mutations may promote immunogenicity in which cancer types. We integrate deleterious somatic and germline mutations and methylation data of DDR genes in 10,080 cancers representing 32 cancer types and evaluate the associations of these alterations with tumor neoantigens and immune infiltrates. Our analyses identify DDR pathway mutations that are associated with higher neoantigen loads, adaptive immune markers, and survival outcomes of immune checkpoint inhibitor-treated animal models and patients. Different immune phenotypes are associated with distinct types of DDR deficiency, depending on the cancer type context. The comprehensive catalog of immune response-associated DDR deficiency may explain variations in immunotherapy outcomes across DDR-deficient cancers and facilitate the development of genomic biomarkers for immunotherapy.

INTRODUCTION

DNA damage repair (DDR) deficiency (DDR-d) leads to increased somatic mutations and accumulation of intracellular DNA fragments that trigger antiviral immune signals. Some of the mutations could function as tumor neoantigens and induce an antitumor immune response.[1] Thus, DDR-d tumors were assumed to show increased immune infiltration and may be particularly susceptible to immune checkpoint inhibitor (ICI) therapy. While significant improvements in response to ICI were observed in subsets of DDR-d tumors,[2,3] roughly half of the DDR-d patients do not benefit from immunotherapy. It remains unclear which DDR-d tumors may respond to ICI in which cancer types, impeding the development of potential biomarkers.

DDR involves many genes that are organized into distinct repair pathways, including damage sensors, single-strand repair processes (base excision repair [BER], nucleotide repair [NER], mismatch repair [MMR]), and double-strand repair mechanisms (non-homologous end joining [NHEJ], microhomology-mediated end joining [MMEJ], and homologous recombination [HR]). The relationship between DDR-d and response to immunotherapy is an active area of research in clinical trials.[4] The US Food and Drug Administration (FDA) has approved pembrolizumab (anti-PD1 therapeutic antibody) to treat MMR-deficient (MMR-d) tumors, as well as cancers with high tumor mutation burden (TMB), regardless of histologic origin.[5] However, while other DDR-ds may also give rise to immunogenic tumors,[4–8] the effectiveness of immunotherapy in other forms of DDR-ds is not yet clear. Furthermore, DDR-ds may arise through pathogenic germline variants (e.g., inherited *BRCA1/2* variants), somatic mutations, or epigenetic silencing (methylation) of genes involved in DNA repair.[9–11] How different types of DDR impairment influence the tumor immune microenvironment remain largely unknown.

While both MMR-d and high TMB status can be used to select a patient for immune checkpoint therapy, these predictive markers are far from perfect.[4] First, MMR deficiency is generally confined to a fraction of tumors within a few cancer types (e.g., MMR to colorectal and endometrial cancers).[4] Second, high TMB is associated only with improved survival of selected cancer types and cohorts (e.g., head and neck cancer, non-small cell lung cancer), and the criteria that define a high TMB status

This is an open access article under the CC BY license (http://creativecommons.org/licenses/by/4.0/).

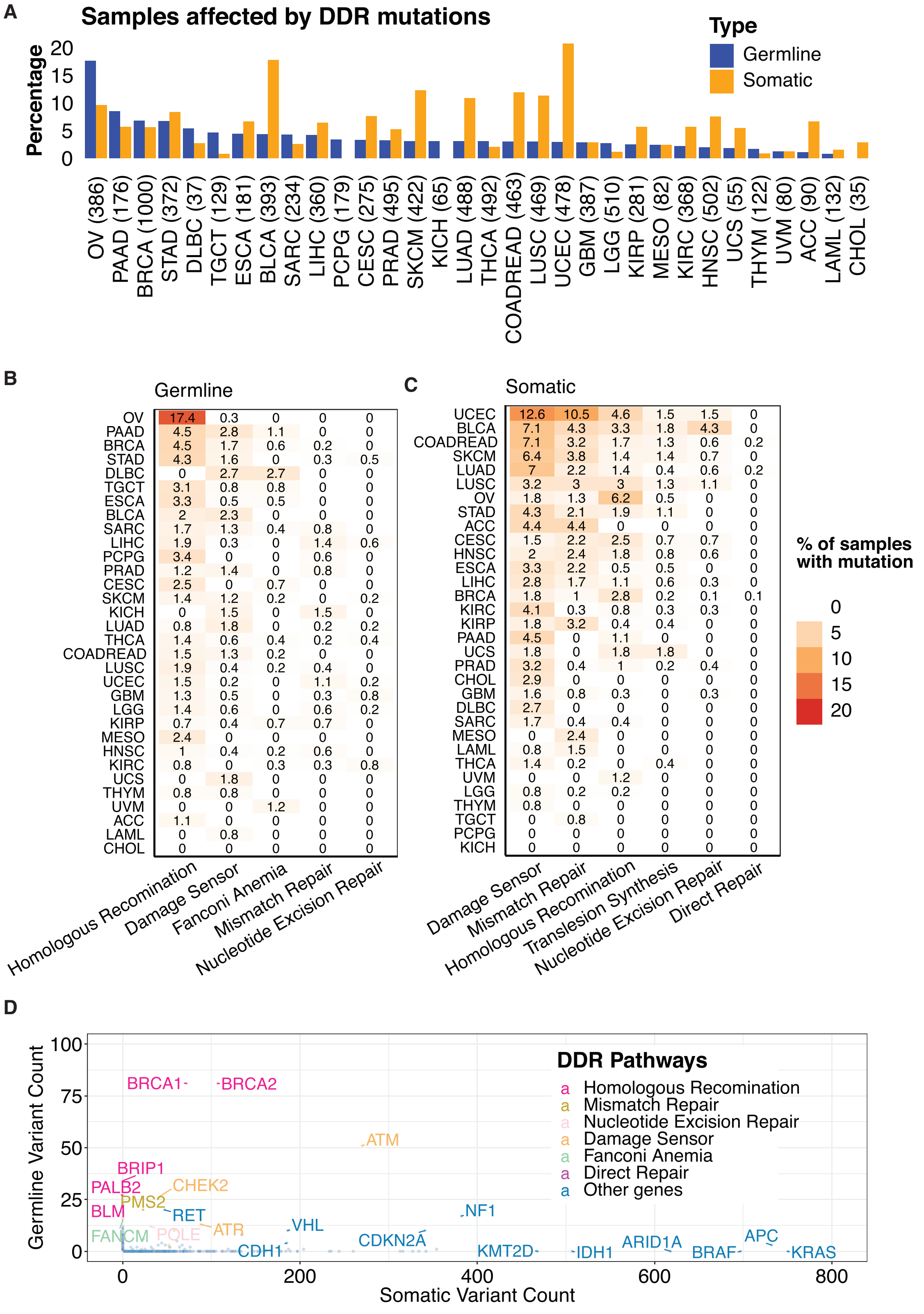

(legend on next page)

CellPress
OPEN ACCESS

remain controversial.[12] Third, while both MMR-d and high TMB are associated with higher immunotherapy response rates, ≥50% of these patients do not respond, highlighting the incomplete understanding of biological processes that determine response.[13,14] Many other DDR genes not included in the classical MMR-d panel are frequently mutated in various cancer types.[11] Elucidating the interactions between the different forms of DDR-d and the immune cell composition of the affected cancer could identify therapeutic opportunities.[9]

The goal of this study was to investigate the relationship between various forms of DDR-d and tumor neoantigen loads, tumor immune infiltration, and further explain the diverse immunogenicity of DDR-d tumors and how those associations correlated with ICI treatment outcomes in different cancer types. We studied the effects of germline pathogenic/likely pathogenic variants, somatic driver mutations of 80 DDR genes in 9,738 non-hypermutated and 342 hypermutated cases, and DNA methylation-mediated silencing of MMR genes. Germline and somatic mutations across (i.e., MMR versus HR) and within (i.e., *BRCA1* versus *BRCA2*) DDR pathways showed variable associations with TMB, neoantigen loads, and indel neoantigen hotspots in a cancer-dependent manner. Several somatic mutations of DDR pathways, but not germline variants, were significantly associated with increased immune infiltration. Deficiencies of different MMR genes showed cancer-specific immune response, and we further showed in a murine model of hepatocellular carcinoma that *MLH1* knockout combined with forced expression of tumor neoantigens improved the survival of mice. Finally, we demonstrated the association between patient survival after ICI therapy and tumor immune infiltrate-associated DDR-ds. Overall, these results identified immune response-associated DDR-ds in a cancer-specific manner, enabling better predictions of ICI response.

RESULTS

Germline and somatic DDR mutations in TCGA non-hypermutated cases

We obtained germline and somatic mutation data and immune gene expression results for 10,080 cancers representing 32 cancer types included in the PanCanAtlas projects in The Cancer Genome Atlas (TCGA; Table S1).[10,15,16] The cancers were categorized into 9,738 non-hypermutated and 342 hypermutated groups[15] that were analyzed separately because their quantitatively and qualitatively distinct genome damage could confound associations with immune phenotypes (Method details). The non-hypermutated cases harbored 783 germline cancer predisposing variants (including pathogenic and likely pathogenic variants, abbreviated as germline variants) and 28,179 somatic driver mutations (abbreviated as somatic mutations) according to the prioritized mutation calls from the PanCanAtlas.[10,15]

In all of the subsequent analyses, we focused on 80 genes involved in HR, NER, and MMR that were designated as core DDR genes by the PanCanAtlas DDR project (Table S2).[11] We found that 4.1% and 7.2% of the 9,738 non-hypermutated cancers harbored germline variants and somatic mutations in these 80 genes, respectively. The germline variant and somatic mutation frequencies differed across cancer types; ovarian cancer (OV) showed the highest frequency (17.6%; cases may carry multiple DDR variants) of carrying germline DDR variants, predominantly affecting *BRCA1/2*, while uterine corpus endometrial carcinoma (UCEC) had the highest frequency (20.7%; cases may carry multiple DDR mutations) of somatic DDR mutations (Figure 1A). Most germline variants affected HR genes (e.g., *BRCA1*, *BRCA2, PALB2*) (Figure 1B), while somatic mutations most commonly affected damage sensors (e.g., *ATM*, *ATR*, *CHEK2*) and MMR genes (e.g., *PMS2*) (Figures 1C and 1D).

Associations between DDR mutations with TMB and tumor neoantigen load

High TMB is emerging as a biomarker of immunotherapy, but the sensitivity of high TMB tumors varied by cancer type.[12] We therefore assessed the association between TMB and neoantigen load and germline variants and somatic mutations in the core DDR genes in 32 cancer types using a multivariate linear regression model, correcting for the age of diagnosis and the population genetic background (Method details). Limiting the analyses to non-hypermutated cases and genes with at least 4 carriers within cancer cohorts, we identified 4 and 24 significant positive associations between germline variants and somatic mutations with TMB, respectively (false discovery rate [FDR] < 0.05; Table S3). In the HR pathway, both germline (FDR = 1.1×10^{-4}) and somatic mutations (FDR = 6.9×10^{-4}) of *BRCA1* were significantly associated with higher TMB in breast invasive carcinoma (BRCA) (Figure 2A), but germline variants or somatic mutations in *BRCA2* showed only a non-significant trend for association with higher TMB after adjusting for multiple comparisons (FDR > 0.072, p < 0.029). However, germline variants of *BRCA2* and *PALB2* were significantly associated with elevated TMB in OV (FDR = 0.0034) and stomach adenocarcinomas (STAD, FDR = 0.032), respectively, while germline *BRCA1* variants showed only a non-significant trend for association with higher TMB in OV (FDR = 0.061, p = 0.017). Somatic mutations of MMR genes (*PMS2*, *MLH1*, and *MSH2*) and DNA damage sensors (*ATR*, *ATM*, and *CHEK2*) and *ERCC2* were associated with higher TMB in UCEC, skin cutaneous melanoma (SKCM), BRCA, kidney renal papillary cell carcinoma (KIRP), colorectal adenocarcinoma (COADREAD), and urothelial bladder carcinoma (BLCA) (FDR < 0.05; Figure 2A; Table S3).

Figure 1. The frequencies of germline predisposing variants and somatic driver mutations of DNA damage repair genes in 9,738 non-hypermutator TCGA cases

(A) The frequencies of samples carrying germline predisposing variants or somatic driver mutations in 80 core DDR genes.

(B) Percentage of cases with germline predisposing variants in DDR pathways.

(C) Percentage of cases with somatic driver mutations in DDR pathways.

(D) Comparison of the total numbers of germline predisposing variants and somatic driver mutations in DDR genes across 9,738 non-hypermutator TCGA cases. Colors represent different DDR pathways.

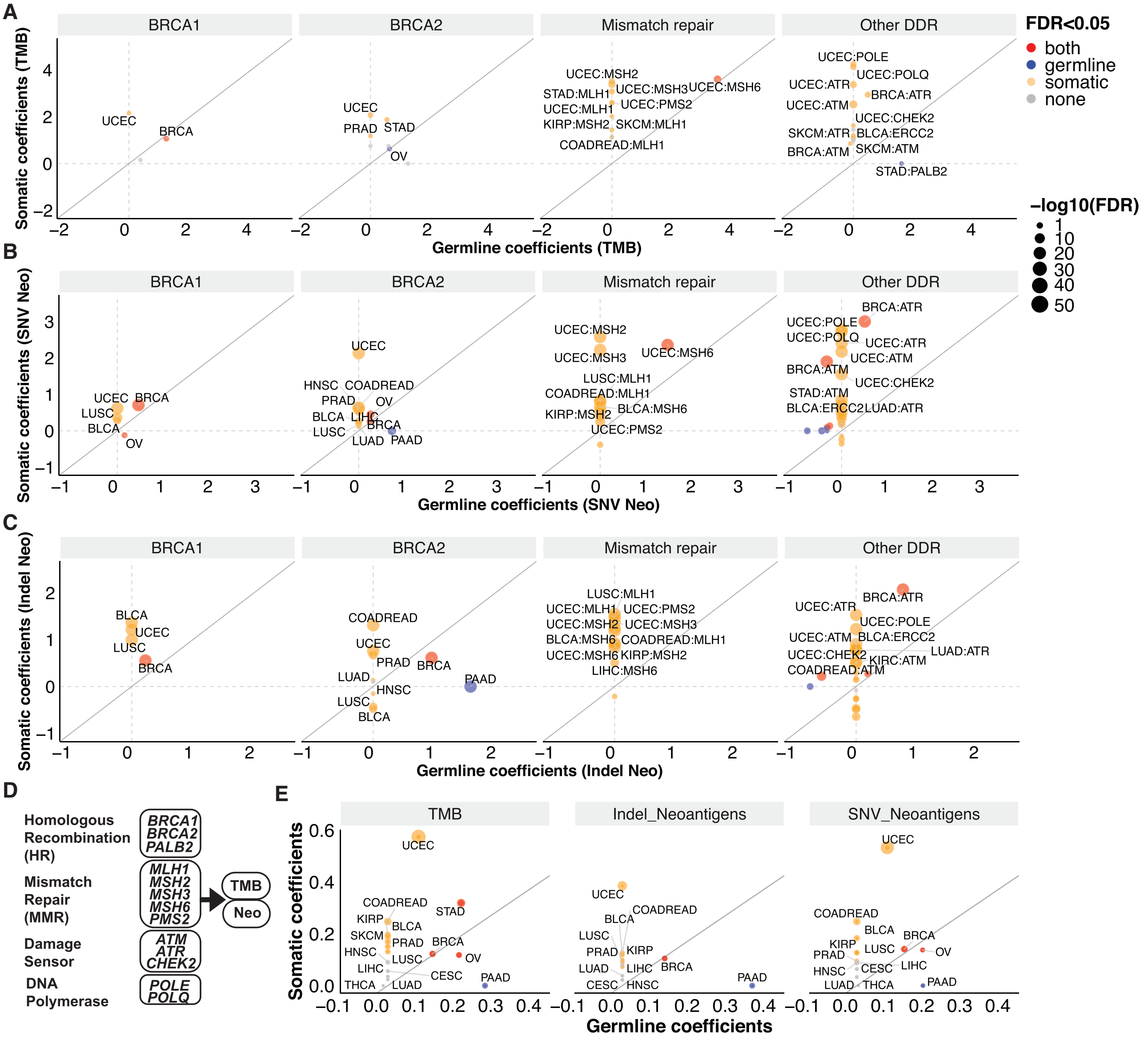

Figure 2. The associations between germline and somatic mutations of DNA damage repair genes with tumor mutation burden (TMB) and neoantigen loads in non-hypermutated cases

(A) Germline and somatic association with TMB.

(B) Germline and somatic associations with SNV neoantigen loads.

(C) Germline and somatic association with indel neoantigen loads. The axes represent the coefficient obtained from the multivariate linear regression analyses.

(D) A schema showing the 13 prioritized DDR genes, which were aggregated into 4 pathways for estimating the combined germline and somatic DDR associations with TMB and neoantigen loads.

(E) The combinatorial germline and somatic DDR associations with TMB and neoantigen loads. The axes represent the coefficients from PLS-PM analysis.

For (A)–(C), and (E), each dot represents a cancer type. Red, blue, orange, and gray represent cancer types in the germline level, somatic level, both levels, and neither level meeting the significance criteria of FDR < 0.05, respectively. The size of the dots represents $-\log_{10}$(FDR), which showed the more significant FDR of either germline or somatic association. The solid gray line indicates equal germline versus somatic associations where the slope = 1.

Germline *MSH6* was also associated with elevated TMB in UCEC (FDR = 0.0022). Overall, germline and somatic mutations in HR genes showed associations with elevated TMB in BRCA, OV, and STAD, although to a variable extent. In UCEC, SKCM, and COADREAD, somatic mutations in MMR and damage sensor genes showed the strongest associations with high TMB. These results indicate varying levels of functional impotence for distinct DDR pathways in maintaining genome integrity in different cancer types.

We next assessed associations between neoantigen load and DDR gene alterations. Fourteen germline alterations (8 genes) and 42 somatic alterations (13 genes) were significantly

associated with elevated SNV-mediated neoantigen load, while 7 germline alterations (5 genes) and 47 somatic alterations (13 genes) were associated with elevated indel-mediated neoantigen load (FDR < 0.05; Table S3) in non-hypermutated cases. Germline and somatic *BRCA1/2* and *ATR* were each significantly associated with higher SNV and indel neoantigen loads in breast cancer (FDR < 1.4×10^{-7}; Figures 2B and 2C). Higher SNV neoantigen loads were found in the germline (FDR = 1.5×10^{-9}) and somatic (FDR = 6.7×10^{-15}) *BRCA2* carriers in OV. We also found that germline *BRCA2* variants were associated with higher SNV and indel neoantigen loads in pancreatic adenocarcinoma (PAAD, FDR = 2.9×10^{-18}, < 1.0×10^{-50}). Somatic mutations of multiple MMR genes as well as *POLE*, *POLQ*, *ATM*, and *ATR* were significantly associated with higher neoantigen loads in UCEC, BRCA, lung adenocarcinoma (LUAD), and STAD (FDR < 1.2×10^{-5}). Germline *MSH6* was associated with higher SNV neoantigens in UCEC (FDR < 1.0×10^{-50}). *MLH1* mutation was strongly associated with high SNV and indel neoantigen load in lung squamous cell carcinoma (LUSC), UCEC, and COADREAD (FDR < 1.0×10^{-28}). Surprisingly, we also found associations with lower neoantigen loads for DDR alterations, including germline *ATM* variants in BRCA, prostate adenocarcinoma (PRAD), LUAD, and BLCA (FDR < 0.011; Table S3), and somatic *BRCA1*, *ATM*, and *ATR* mutations in OV, liver hepatocellular carcinoma (LIHC), and COADREAD (FDR < 0.043; Figures 2B and 2C; Table S3), respectively. Overall, the patterns of associations of TMB and neoantigen load with germline and somatic mutations in core DDR genes indicate a strong similarity; the minor discordances suggest either noise in the data or distinct DNA damage profiles induced by different types of DDR-ds.

We adopted the partial least-squares path modeling (PLS-PM) method to dissect the combinatorial effect of germline and somatic DDR mutations on TMB and neoantigen load. The PLS-PM model was constructed with the 13 DDR genes associated with higher TMB/neoantigen loads (germline or somatic regression coefficient > 1.5 and FDR < 0.05 in any cancer type), including multiple HR (*BRCA1/2*, *PALB2*), MMR (*MLH1*, *MSH2*, *MSH3*, *MSH6*, and *PMS2*), damage sensor (*ATM*, *ATR*, and *CHEK2*), and DNA polymerase (*POLE*, *POLQ*) genes (Figure 2D). These 4 DDR pathways (13 genes) were also investigated for their association with immune infiltration in the tumor microenvironment in subsequent sections. We introduced two latent variables representing the combined effects of (1) germline-affected genes and (2) somatic-affected genes in the PLS-PM analysis and estimated their relative contributions to TMB and neoantigen load (Figure S1). PLS-PM revealed cancer types displaying variable associations between germline and somatic DDR associations with TMB and neoantigen loads (Figure 2E). In BRCA and OV, germline variants (FDR < 0.0068) and somatic mutations (FDR < 0.012) showed a similar, independent contribution to TMB and neoantigen loads. Strong associations between germline variants (FDR = 0.0012) and somatic mutations (FDR = 1.3×10^{-9}) and TMB were identified in STAD. In PAAD, germline variants (FDR < 0.0030) but not somatic mutations (FDR = 1.0) were significantly associated with elevated TMB and neoantigen loads. For other, non *BRCA*-associated cancer types, such as UCEC, COADREAD, BLCA, and KIRP, somatic mutations were associated with higher TMB and neoantigen loads (FDR < 0.0020), while germline variants showed minimal associations (Figure 2E), potentially due to their rarity or limited functional consequences in those cancer types.

DDR pathway-level alterations associated with tumor neoantigens load and hotspots

Previous results suggested that mutations affecting DDR genes within the same pathway frequently showed similar effects on genome damages across cancer types, and thus, grouping these genes by functional pathways may aid discovery in cohorts with limited mutated cases. The 13 DDR genes associated with higher TMB/neoantigen loads were grouped into 4 core pathway annotations, as shown in Figure 2D. Based on this classification of DDR-ds, we examined how neoantigen loads may be associated with germline variants and somatic mutations affecting DDR genes. Carriers of somatic MMR mutations showed higher predicted SNV neoantigen loads in UCEC, COADREAD, cervical squamous cell carcinoma and endocervical adenocarcinoma (CESC), and BLCA (FDR < 0.037; Figure 3A) and higher indel neoantigen loads in UCEC (FDR = 1.2×10^{-9}; Figure 3B). Somatic mutations of HR genes are associated with higher indel neoantigen loads in UCEC, BRCA, and COADREAD, as well as higher SNV neoantigen loads in the same cancer types and BLCA (FDR < 0.047). Germline HR genes were only associated with higher SNVs (FDR = 3.0×10^{-5}) and indel neoantigen loads in BRCA (FDR = 0.0068; Figures 3A and 3B). Somatic mutations of damage sensor genes were associated with increased SNV (FDR = 1.6×10^{-11}) and indel (FDR = 1.2×10^{-7}) neoantigen loads in UCEC (Figures 3A and 3B). Somatic mutations of DNA polymerases were associated with increased SNV neoantigen loads of UCEC (FDR = 2.1×10^{-4}) and CESC (FDR = 0.036; Figure 3B). No significant association was identified in other cancer types.

While neoantigens are commonly found in hypermutated MSI tumors, whether non-hypermutated tumors may harbor potentially targetable neoantigens remains less characterized. We further investigated the associations of DDR mutations with 11 SNV and 178 indel neoantigen "hotspots" found in ≥5% of TCGA non-hypermutated cases (Method details) in the pan-cancer, non-hypermutator cohort. A total of 56 (of 178) indel neoantigen hotspots were significantly (FDR < 0.05) associated with the DDR mutations (Figure 3C), whereas no enrichment of SNV hotspots was identified. For example, *DAZAP1* p.P257Rfs*78, *DOCK3* p.P1852Qfs*45, and *RNF43* p.G659Vfs*41 were each highly enriched in cases with somatic MMR mutations (FDR < 2.1×10^{-6}) and *DAZAP1* p.P257Rfs*78 was also associated with germline MMR variants (Figure 3C; FDR = 0.0024). Notably, *RNF43* p.G659Vfs*41 has been shown to be an expressed, immunogenic neoantigen associated with ICI-treated patient outcomes.[17,18] The enrichment of neoantigens associated with non-hypermutated DDR-d cases suggests the possibility of recognition by adaptive immunity, and their immunogenicity warrants further investigation.

Immune infiltration associated with DDR-d

Within each cancer type, we compared gene expression-based measures of adaptive immune response between cancers affected by either germline or somatic alterations in DDR

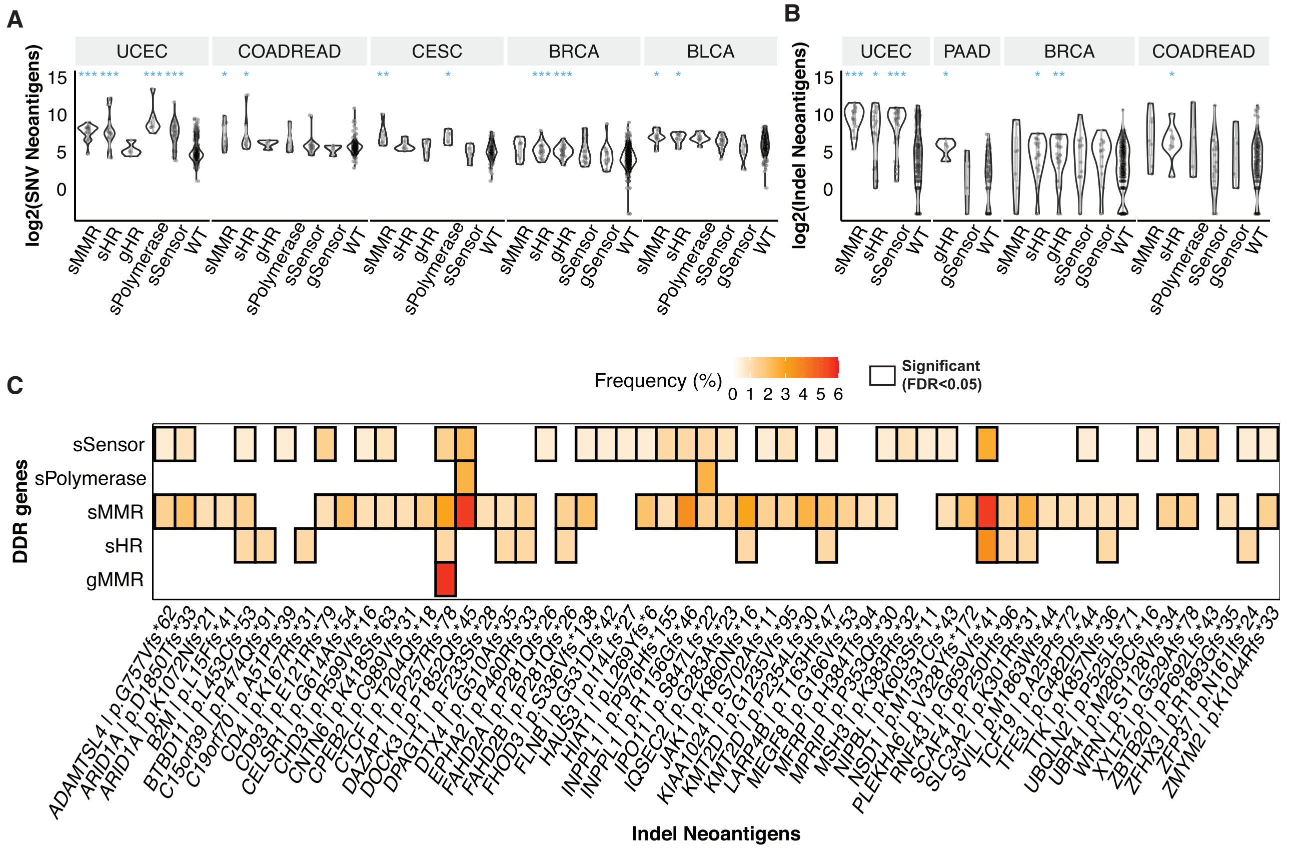

Figure 3. The association between DNA damage repair pathway mutations and tumor neoantigens in non-hypermutated cases

(A) The distribution of SNV neoantigen loads in DDR mutant samples.

(B) The distribution of indel neoantigen loads in DDR mutant samples. Two-sided Wilcoxon rank-sum tests were applied to compare each mutant group with DDR-wild-type (WT) cases and FDR adjusted. FDR < *0.01, **0.001, and ***0.0001.

(C) The enrichment of neoantigen hotspots in DDR mutant samples in the pan-cancer cohort. The heatmap shows the significant (FDR < 0.05, black box) enrichment of neoantigens in DDR mutant samples. The value and color in each cell represent the percentage of cases with the listed neoantigen in DDR mutant cases. DDR pathways annotated with prefix "s" indicate somatic mutations, and prefix "g" indicates germline variants in the DDR pathways, including mismatch repair (MMR) (*MLH1*, *MSH2*, *MSH3*, *MSH6*, and *PMS2*), homologous recombination (HR) (*BRCA1/2* and *PALB2*), sensor (*ATM*, *ATR*, and *CHEK2*) and polymerase (*POLE* and *POLQ*).

pathways (MUT [mutated]) versus cancers without alterations in these genes (WT [wild type]) using a multivariate regression model corrected for demographic variables in 9,738 non-hypermutated cancers (Method details). We identified 7 positive associations (FDR < 0.05) between the somatic mutations in DDR pathways with tumor-infiltrating lymphocytes (TILs, based on hematoxylin and eosin [H&E] stained tissue image analysis)[16] and immune gene expression measures, including *PD1/PD-L1* expression, and the high cytolytic activity (CYT) score[19] in UCEC (Table S4). None of the germline DDR pathway alterations were significantly associated with immune signatures (FDR > 0.43; Table S4). Somatic mutations of the MMR pathway were associated with higher TILs, CYT scores, and *PD1/PD-L1* expression in UCEC (FDR < 0.011; Figures 4A–4D). UCEC cases with damage sensor somatic mutations showed higher CYT scores (FDR = 0.031; Figure 4B) and higher *PD1* expression (FDR = 0.034; Figure 4C). Somatic mutations of DNA polymerase in UCEC were associated with higher *PD-L1* expression (FDR = 0.049; Figure 4D). We also conducted a gene-level analysis using the multivariate model. None of the germline DDR genes were associated with immune signatures, and somatic mutations in *MSH6*, *ATM*, and *MSH2* were associated with increased immune response in UCEC (Table S5; FDR < 0.042).

To evaluate the concordance between DDR-associated increased neoantigen load and higher immune infiltration, we compared the correlation coefficients obtained from neoantigen load and immune signature analyses (Method details). The associations of somatic DDR mutation versus SNV neoantigen loads were highly correlated with the association of somatic DDR mutation versus immune signatures (TILs, CYT score, *PD1* expression, and *PD-L1* expression) ($r > 0.46$, $p < 9.4 \times 10^{-4}$; Figures 4E–4H). For indel neoantigen loads, we observed a similar positive correlation between DDR versus indel neoantigen and DDR versus immune signatures ($r > 0.50$, $p < 0.001$; Figures 4I–4L) after removing an extremely low linear regression coefficient (−4.33, from damage sensor mutations in sarcoma

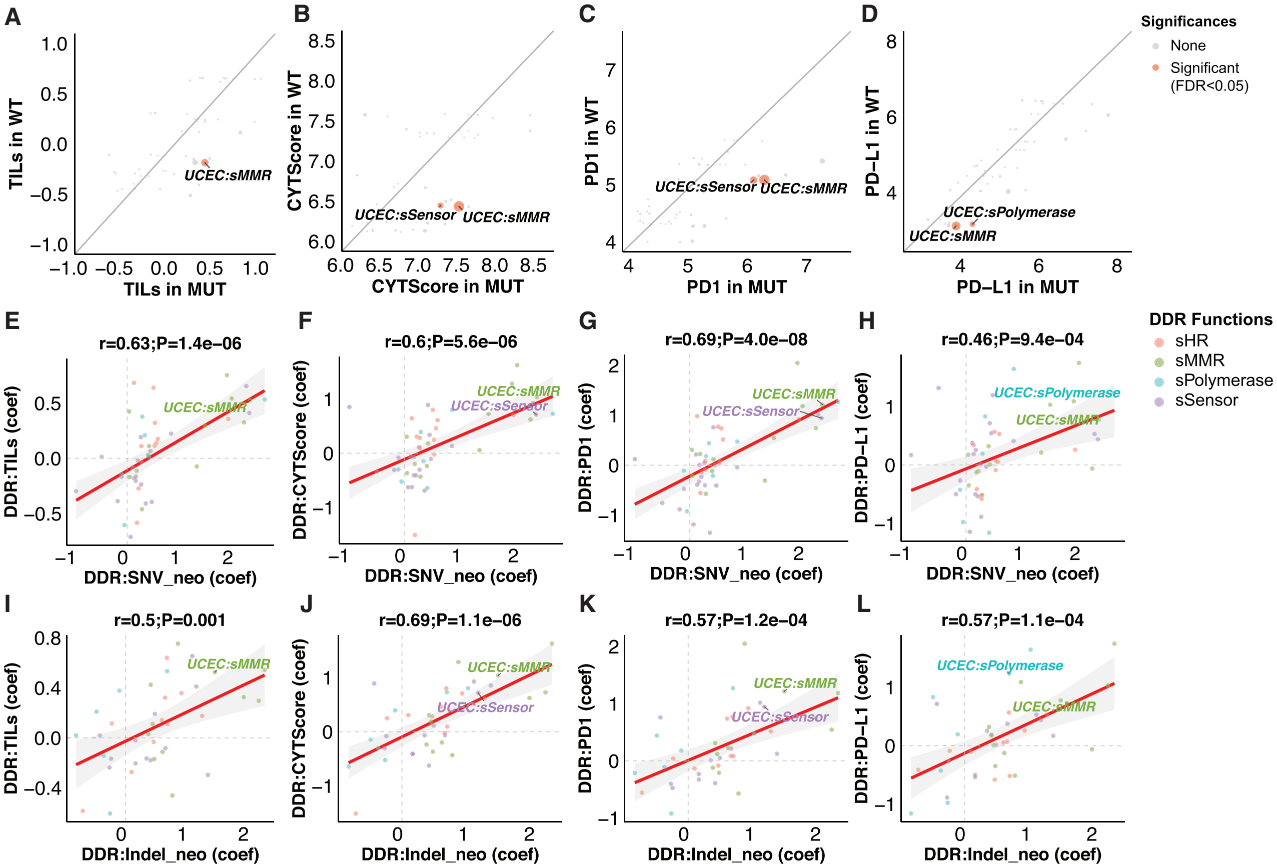

Figure 4. The association between DNA damage repair mutations and tumor immune infiltrates in non-hypermutated cases

(A–D) Comparisons of the tumor immune infiltrate signatures in germline and somatic DDR mutated (MUT) and WT cases, including (A) tumor-infiltrating lymphocytes (TILs), (B) cytolytic activity (CYT) score, (C) *PD1* expression, and (D) *PD-L1* expression. Significance values were generated by linear regression adjusted by patients' age and genetic principal components and FDR corrected. Gray, blue, and red represent cancer types that meet the criteria of FDR < 0.05 (significant) and FDR > 0.05 (none). The size of the dots represents $-\log_{10}$(FDR).

(E–H) Comparisons of the correlation coefficients of somatic DDR versus SNV neoantigen load with the somatic DDR versus immune signature, including (E) TILs, (F) CYT score, (G) *PD1* expression, and (H) *PD-L1* expression.

(I–L) Comparison of the correlation coefficients of somatic DDR versus indel neoantigen load with the somatic DDR versus immune signature, including (I) TILs, (J) CYT score, (K) *PD1* expression, and (L) *PD-L1* expression.

r represents the Pearson correlation coefficient. The color of the label represents the DDR pathways, including MMR (*MLH1*, *MSH2*, *MSH3*, *MSH6*, and *PMS2*), HR (*BRCA1/2* and *PALB2*), sensor (*ATM*, *ATR*, and *CHEK2*) and polymerase (*POLE* and *POLQ*). DDR genes annotated with prefix "s" indicate somatic mutations. The axes indicate the correlation coefficients (coef) of linear regression adjusted by patients' age and genetic components. Dots with significant associations between DDR mutations and immune signatures were labeled in all of the panels.

[SARC]). Similar correlations were not observed for germline DDR variants (Figure S2). These results suggest that somatic DDR-ds can be predictive of tumor immune infiltration, at least partially through its association with greater neoantigen load.

Somatic DDR mutations associated with hypermutators and microsatellite instability

DDR mutations, particularly those disrupting MMR, are thought to drive microsatellite instability (MSI) and hypermutator phenotypes.[20] We investigated how DDR mutations may be associated with the hypermutator phenotype and MSI. Among the 342 hypermutator samples (3.4% of 10,080 TCGA cases),[15] 63.7% carried at least 1 somatic mutation of 80 DDR genes, 2.9% carried at least 1 germline variant, and 5.8% had both germline and somatic alterations. At the gene level, somatic mutations of *ATM* (22.2%) were most common in hypermutators, followed by somatic *BRCA2* (17.8%), *POLQ* (10.8%), and *ATR* (10.5%) (Figure S3A). Somatic mutations of MMR genes, such as *MSH2*, *MLH1*, *MSH6*, *MSH3*, and *PMS2*, were also found in 31.3% of hypermutators. We further analyzed the association between mutation of 13 prioritized DDR genes and hypermutator status within each cancer type, identifying 38 significant associations (FDR < 0.05; Figure S3B). Somatic mutations of multiple DDR genes are associated with hypermutators of UCEC, COAD-READ, and STAD. Germline *BRCA1* variants were associated with the hypermutated BRCA cases (FDR = 0.048; Figure S3B).

We also examined the associations between DDR genes with MSISensor scores,[21] a measure of MSI derived from the

whole-exome sequencing data. As expected, tumors with germline variants or somatic mutations of the 13 prioritized DDR genes had higher MSISensor scores than non-carrier cases in UCEC, STAD, COADREAD, BRCA, and CESC (FDR < 0.0035; Figure S3C). We next applied the germline/somatic PLS-PM multivariate model to estimate the relative associations of DDR genes and MSI scores. In the models for UCEC, STAD, and COADREAD, somatic mutations were significantly associated with MSI scores (FDR < 1.20×10^{-2}; Figures S3D–S3F). At the gene level, *POLE* showed the highest contributions to the somatic latent variable in STAD (β = 0.61; Figure S3E) and COADREAD (β = 0.62; Figure S3F), while also contributing strongly in UCEC (β = 0.46; Figure S3D). *MSH3* showed the top somatic contribution in UCEC (β = 0.59; Figure S3D) and was the third contributor in STAD (β = 0.53; Figure S3E). These results revealed the varied effect sizes on MSI associated with different DDR mutations, which may result in different levels of neoantigens and tumor immune response.

MMR deficiency affects tumor immune infiltrates in a cancer-specific manner

Although mutations of four MMR genes (*MLH1*, *MSH2*, *MSH6*, and *PMS2*) were incorporated into FDA-approved indications for ICIs, over half of MMR-deficient tumors still do not respond to immunotherapy,[13,14] suggesting variable immunogenicity across cases. We investigated the tumor neoantigens and immune infiltrates in cases affected by germline variants/somatic mutations (mut) and/or methylation (me) of 5 MMR genes: *MLH1*, *MSH3*, *MSH2*, *MSH6*, and *PMS2*. In the pan-cancer cohort of 10,080 cases (hypermutators and non-hypermutators), the frequencies of MMR-d cases, affected MMR genes, and alteration types varied across cancer types (Figure 5A). UCEC had the highest portion of cases carrying MMR mutations and/or methylation (36.5%), followed by 18.9% in STAD and 15.0% in COADREAD. Notably, methylation of the *MLH1* gene was the most common MMR alteration, affecting 2.7% of total TCGA cases. Mutations of *MSH2*, *MSH6*, and *PMS2* affects 0.8%, 0.9%, and 0.6% of TCGA cases, respectively (Figure 5A).

We compared indel and SNV neoantigen loads in the MMR gene carriers (including any of germline variants, somatic mutations, and methylations) versus non-carriers (WT) within cancer types (Figures 5B and 5C). Given the prevalence of co-occurring alterations in MMR genes, we tested all single- and pair-gene alterations with at least four affected cases in one cancer cohort. Cases with *MLH1* alterations showed higher SNV neoantigens in UCEC, COADREAD, STAD, CESC, BLCA, and LUSC (FDR < 0.044; Figure 5B) and associated with higher indel neoantigen loads in UCEC, COADREAD, and CESC (FDR < 0.014; Figure 5C). *MSH2* mutations were associated with higher SNV/indel neoantigen loads in UCEC and COADREAD (FDR < 0.022). *MSH2* alterations co-occurring with *MSH3 and MSH6* were associated with increased neoantigen loads in UCEC (FDR < 0.011). *MSH6* mutations were associated with higher SNV neoantigen loads in UCEC, COADREAD, and BLCA (FDR < 0.010) and higher indel neoantigen loads in UCEC (FDR = 0.0026; Figures 5B and 5C).

To delineate their independent associations, we applied a multivariate regression model that includes both mutation and methylation of *MLH1*, *MSH3*, *MSH2*, *MSH6*, and *PMS2* genes as predictors of immune signatures, including TILs, *PD1*, *PD-L1* expression, and CYT score in COADREAD, STAD, and UCEC (Figure 5D). For COADREAD, *MLH1* methylation was significantly associated with TILs, *PD1* expression, *PD-L1* expression, and CYT score (FDR = 0.029, 1.1×10^{-5}, 2.5×10^{-13}, and 9.6×10^{-13}, respectively). For STAD, *MLH1* methylation was positively associated with *PD-L1* expression (FDR = 0.0011) and CYT score (FDR = 0.020). For UCEC, *MSH2* mutations were positively correlated with *PD1* expression (FDR = 0.011) and CYT score (FDR = 0.0093). Mutations of *MSH6* were also positively associated with TILs, *PD1* expression, *PD-L1* expression, and CYT score (FDR = 0.00078, 6.0×10^{-5}, 0.0025, 3.2×10^{-6}, respectively) in UCEC (Figure 5D). These results delineated the cancer-specific immunogenic effects of MMR genes that may require further considerations for their biomarker applications.

The combinatorial effect of *MLH1* deficiency and neoantigen loads

The aforementioned results suggested a dominant immunogenic effect of *MLH1* deficiency induced by methylation or mutations in COADREAD. We further stratified COADREAD cases into those with high/low SNV or indel neoantigen loads based on the respective average values dividing the data distribution (Figure S4A). As expected, most *MLH1*-deficient cases were enriched in the groups of high SNV (93.1%, odds ratio = 46.5, $p < 2.2 \times 10^{-6}$) and indel (92.7%, odds ratio = 19.1, $p = 1.5 \times 10^{-14}$) neoantigen loads (Figure S4B). However, *MLH1* deficiency and high neoantigen loads do not always co-occur, and it remains unclear whether *MLH1* status can further stratify tumor immunogenicity beyond neoantigen loads. We investigated the distribution of immune signatures in COADREAD patients stratified by *MLH1* deficiency and neoantigen load, including *MLH1* deficiency/high neoantigen load, *MLH1*-WT/high neoantigen load, *MLH1* deficiency/low neoantigen load, and *MLH1*-WT/low neoantigen load. As expected, cases with both *MLH1* deficiency/high neoantigen load also exhibited the highest immune signatures, including *PD1*/*PD-L1* expression and TILs (Figures 6A–6H). For *MLH1*-WT cases, high SNV neoantigen loads were also commonly associated with elevated immune response signatures (Figure 6A–6D), whereas high indel neoantigen load was only significantly associated with increased CYT score (Figure 6F). Critically, in most cases, *MLH1* deficiency further stratified neoantigen-high tumors for significantly higher immunogenicity.

Previous reports have demonstrated increased immune surveillance upon the inactivation of *MLH1* in syngeneic mouse models of colorectal cancer.[22] Given that MMR-d is now FDA approved across cancer types as a biomarker for ICI, understanding its effect in a microsatellite stable (MSS) cancer type would be critical to broaden the potential application of ICI. We experimentally tested whether *MLH1* deficiency could confer a survival benefit in autochthonous MYC;sg-$p53^{-/-}$ hepatocellular carcinoma (HCC) murine tumors. This model includes a transposon-based vector to overexpress oncogene *MYC* and a CRISPR-based vector to delete tumor suppressor *p53*. To mimic immunogenic neoantigen expression, the *MYC* overexpression vector was modified

CellPress
OPEN ACCESS

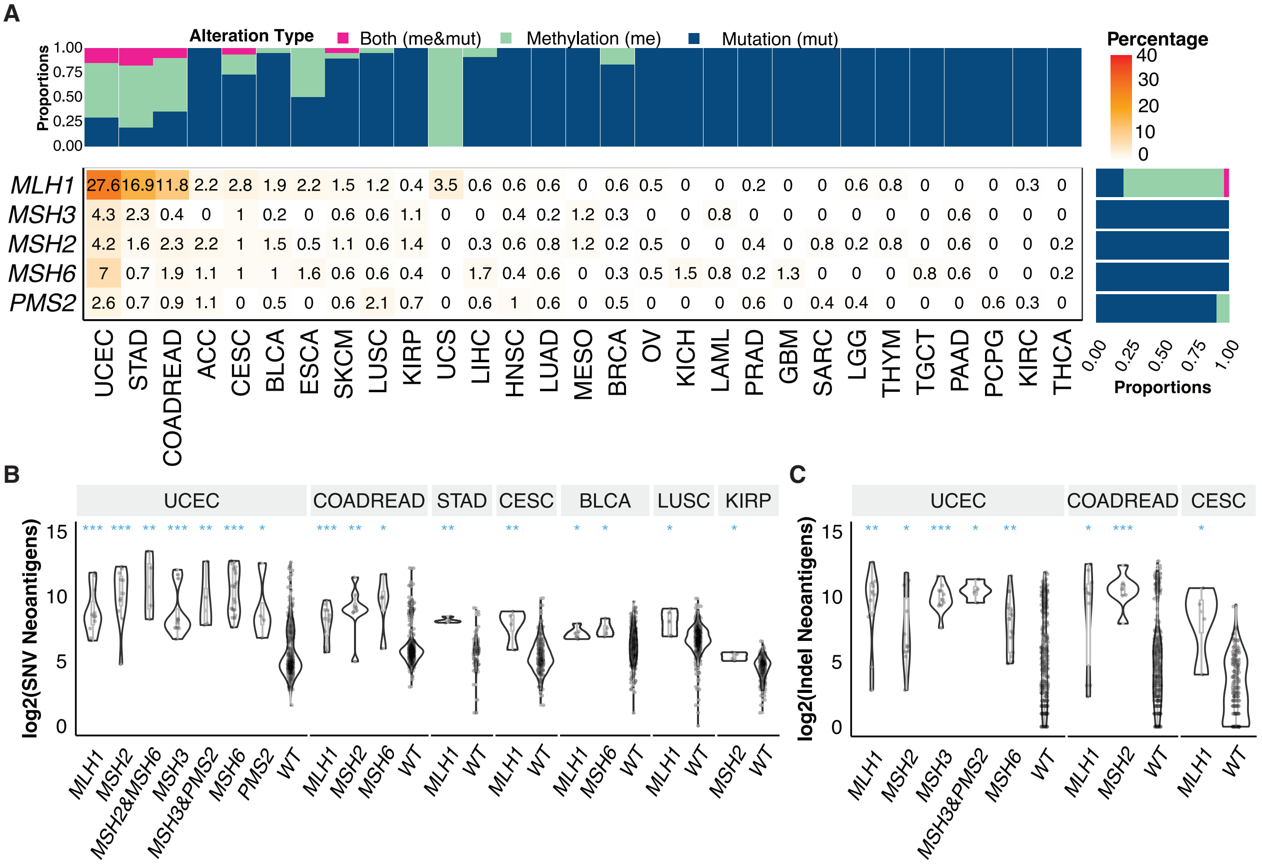

D

Cancer	Immune	Immune signature~mutMLH1+meMLH1+mutMSH3+mutMSH2+ mutMSH6+mutPMS2 +Age+Genetic principal components					
		mutMLH1	***meMLH1***	***mutMSH3***	***mutMSH2***	***mutMSH6***	***mutPMS2***
COAD READ	TILs	0.11	**0.38***	NA	-0.16	0.07	0.75
	PD1	0.65	**1.00*****	NA	-0.23	0.76	1.05
	PD-L1	0.90	**1.54*****	NA	0.27	-0.19	1.40
	CYTScore	0.84	**1.57*****	NA	0.23	0.56	1.06
STAD	TILs	0.16	-0.10	-0.58	0.67	NA	NA
	PD1	0.58	0.39	-0.85	0.76	NA	NA
	PD-L1	0.76	**0.81****	0	0.68	NA	NA
	CYTScore	0.51	**0.60***	-0.15	0.99	NA	NA
UCEC	TILs	0.11	0.10	0.04	0.39	**0.69*****	0.26
	PD1	0.51	0.40	-0.02	**1.28****	**1.47*****	0.63
	PD-L1	0.43	0.06	0.19	0.46	**0.84****	0.44
	CYTScore	0.43	0.20	0.10	**1.07****	**1.37*****	0.44

Figure 5. Genetic and epigenetic alterations of mismatch repair genes in 10,080 non-hypermutated and hypermutated cases

(A) The frequencies of TCGA samples carrying genetic (include germline variants and somatic mutations) and epigenetic alteration of *MLH1*, *MSH3*, *MSH2*, *MSH6*, and *PMS2* genes. The upper bar plot shows the frequency samples, with genetic alterations (mut) and methylation (me) of MMR genes within each cancer type. The right bar plot shows the frequency of genetic alterations and methylation of each of the 6 MMR genes across all 10,080 cases. The heatmap showed the percentage of cases carrying alterations in each MMR gene in each cancer type, where the color and value indicate the frequency (%). Only cancer types with at least 1 carrier of MMR alterations were shown.

(B and C) The neoantigen comparisons between MMR affected and WT cases, including (B) indel neoantigen loads and (C) SNV neoantigen loads. The alterations of each gene shown in the x axis of (B) and (C) include germline variants, somatic mutations, and methylations.

(D) The multivariate model associations of genomic alteration of individual MMR genes and immune signatures, including TILs, *PD1, PD-L1* gene expression, and CYT score. FDR < *0.01, **0.001, and ***0.0001.

Three MMR-d-enriched cancer types, COADREAD, STAD, and UCEC, were included in the analysis of D.

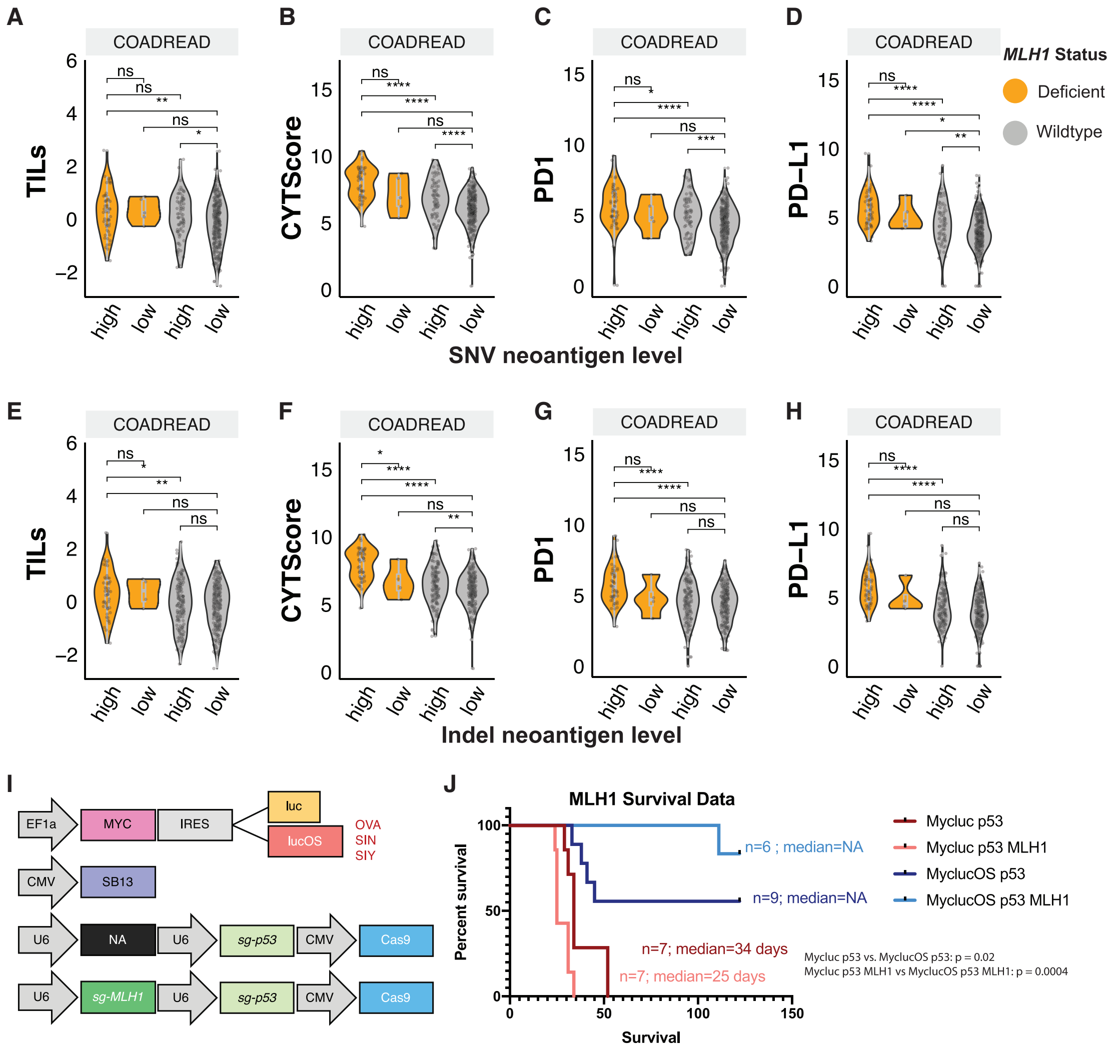

Figure 6. Neoantigen load and immunogenicity of *MLH1*-deficient tumor

(A–D) The distribution of immune signatures, including (A) TILs, (B) CYT score, (C) *PD1*, and (D) *PD-L1* expression, in cases stratified by *MLH1* deficiency (*dMLH1*), *MLH1*-WT (*wtMLH1*), high (h), and low (l) SNV neoantigen load.

(E–H) The distribution of immune signatures, including (E) TILs, (F) CYT score, (G) *PD1*, and (H) *PD-L1* expression, in cases stratified by *dMLH1*, *wtMLH1*, h, and l indel neoantigen load. p values were estimated by the 2-sided Wilcoxon rank-sum test and FDR corrected. FDR < *0.01, **0.001, and ***0.0001. ns, not significant.

(I) Schematic of vectors injected into mice. The transposon-based vector overexpressing *MYC* and luciferase (Mycluc) or a luciferase fused to model antigens (MyclucOS).

(J) The survival rate of mice in each group shown as well as median survival, including *MLH1* WT group (Mycluc p53, n = 7), *MLH1*$^{-}$ group (Mycluc p53 *MLH1*, n = 7), *MLH1* WT + antigens group (MyclucOS p53, n = 9), and *MLH1*$^{-}$ + antigens group (MyclucOS p53 *MLH1*, n = 6). The log-rank Mantel-Cox test was used to calculate the p values.

to also express either luciferase (luc) or a luc fused to three strong murine T cell-activating antigens SIY, SIN, and SIINFEKL (lucOS).[23] To model *Mlh1* deficiency in the context of SIY, SIN, and SIINFEKL expression, we modified the single-guide (sg)-*p53* CRISPR vector to incorporate an sgRNA to target the *Mlh1* gene.[22] We generated 3 separate tandem sg-*p53*;sg-*Mlh1* CRISPR-based vectors, each harboring a unique guide RNA targeting different portions of the *Mlh1* gene (Figure 6I). Using these tools, we created 2 murine models of *Mlh1*-deficient HCC: the non-immunogenic *MYC*-luc;sg-*p53*;sg-*Mlh1* (Mycluc p53 *MLH1*) and the immunogenic counterpart MYC-lucOS;sg-*p53*;sg-*Mlh1* (MyclucOS p53 *MLH1*), which expresses the 3 antigens (Figure 6I). Controls for these conditions were the previously established *MYC*-luc;sg-*p53* (Mycluc p53) and *MYC*-lucO;g-*p53* (MyclucOS p53) mice.[23]

The expression of antigens (lucOS) in the context of *MYC*;sg-*p53*$^{-/-}$ tumors led to a significant delay in tumorigenesis compared to *MYC*-luc;sg-*p53* tumors lacking the antigens (p = 0.021) due to the induction of antitumor immune response, as previously reported.[23] The introduction of *Mlh1* deletion slightly accelerated tumor formation in the absence of exogenous antigen expression. However, in the presence of the antigens

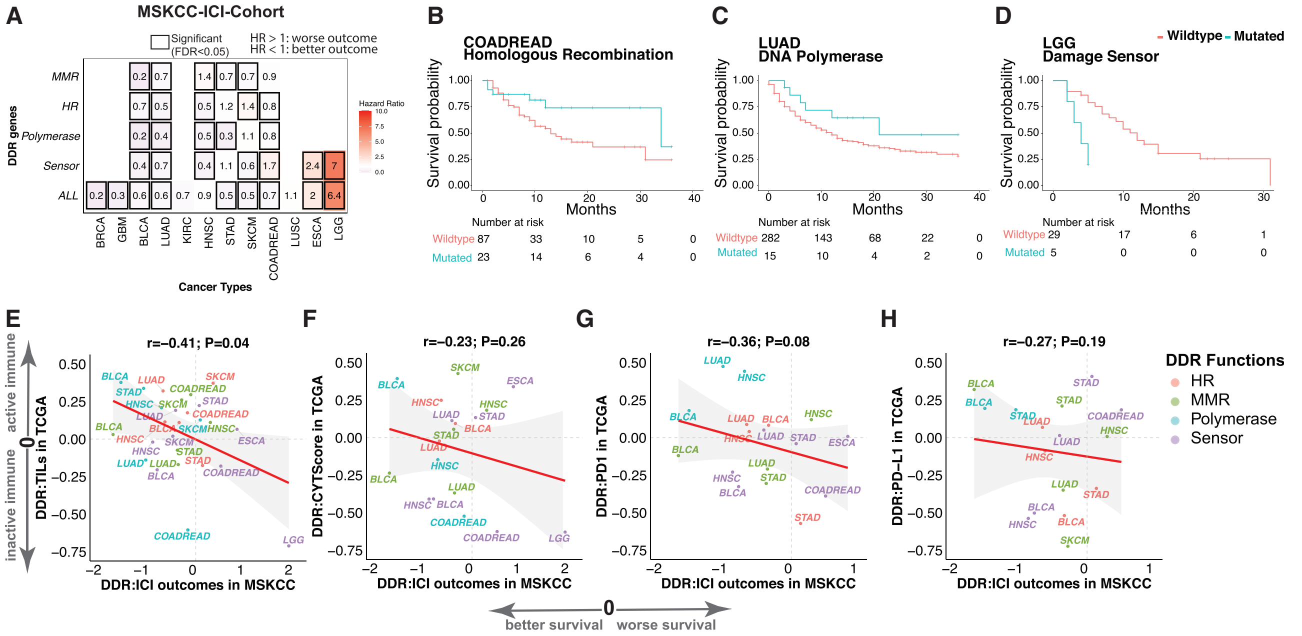

Figure 7. Association between survival outcomes of immune checkpoint inhibitor (ICI)-treated cases and DDR pathway mutations

(A) The heatmap shows the hazard ratio of DDR genes in the MSKCC ICI-treatment cohort. The x axis represents cancer types, and the y axis represents somatic mutations in DDR genes, including MMR, HR, damage sensor (sensor), DNA polymerase (polymerase), and all of the 13 prioritized DDR genes (ALL). The value in each cell denotes the hazard ratio. Hazard ratios >1 and <1 suggest the association of DDR mutations with worse and better survival, respectively. Hazard ratio and p values were calculated using multivariable Cox proportional hazards models. The black box indicates FDR meets the criteria of <0.05 (significant). An empty cell indicates that the analysis was not conducted due to insufficient carrier counts.

(B–D) Survival curve of MSKCC patients with and without somatic mutations in prioritized DDR genes, including (B). HR genes (*BRCA1*, *BRCA2*, and *PALB2*) in COADREAD. (C) Damage sensor genes (*ATM*, *ATR*, and *CHEK2*) in brain lower-grade glioma (LGG), and (D) DNA polymerase genes (*POLE*, *POLQ*) in LUAD.

(E–H) Correlation between the DDR-survival association in the MSKCC cohort versus the DDR-immune infiltrate association in the TCGA cohort, including (E) TILs, (F) CYT score, (G) *PD1* expression, and (H) *PD-L1* expression. *r* represents Pearson correlation coefficient.

(lucOS), it produced a more pronounced antitumor effect (p = 0.0004) (Figure 6J). While the median survival was not reached in either immunogenic condition (MyclucOS p53 and MyclucOS p53 *MLH1*), there was a clear survival advantage in the MyclucOS p53 *MLH1* group compared with the MyclucOS p53 group. Similar to Germano et al.,[22] this observation is consistent with an enhanced antitumor immune response induced by *MLH1* loss. In parallel, MMR-mutated human HCC cases in the TCGA cohort also showed significantly higher *PD-1* gene expression (multivariate regression, p = 0.023, data not shown in the figure), supporting MMR mutations can distinguish immunogenic tumors that may show better survival upon ICI treatment among HCC.

DDR-ds predictive of immunotherapy outcomes

To test the association between DDR-d and immunotherapy outcomes in patients, we obtained somatic mutations and clinical outcomes data from ICI-treated patients at Memorial Sloan Kettering Cancer Center Cancer (MSKCC),[12] which included 12 cancer types totaling 1,525 cases with complete information (Method details). For cohorts with at least 4 mutated cases, we used a multivariate Cox survival model to identify the associations between the 13 prioritized DDR genes and survival outcomes, adjusting for the patient's age, gender, and different ICI drugs. No significant (FDR < 0.05) association was identified at the individual gene level (Figure S5A). We also assessed associations at the pathway level when we grouped the 13 genes into 4 DDR pathways, and at a combined DDR-d level when all 13 genes were considered together.

At the pathway level, we identified 22 significant associations of somatic DDR mutations and survival after immunotherapy in 8 of the 12 cancer types in the clinical data, and 15 (75.0%) of the associations were toward better survival (Figure 7A). For example, somatic mutations of MMR genes were associated with better clinical outcomes in BLCA, LUAD, SKCM, and STAD (FDR < 0.035), but associated with worse survival of head and neck squamous cell carcinoma (HNSC) (FDR = 0.038). Somatic mutations of HR and DNA polymerase were each associated with better survival in COADREAD and LUAD (FDR < 0.026; Figures 7A–7C). Notably, HR mutations were associated with worse survival of SKCM (FDR = 1.3×10^{-6}), while damage sensor mutations were associated with worse outcomes in COADREAD, esophageal carcinoma (ESCA), and lower-grade glioma (LGG) (FDR < 2.2×10^{-4}; Figures 7A and 7D). Cases carrying mutations in any of the 13 prioritized DDR genes (ALL), showed improved outcomes in BRCA, glioblastoma (GBM), BLCA, LUAD, STAD, SKCM, and COADREAD (FDR < 1.4×10^{-5}), but worse outcomes in ESCA and LGG (FDR = 0.0013 and 1.7×10^{-19}, respectively) (Figure 7A). Compared to BLCA, LUAD, and STAD, the mix of positive and negative treatment outcomes found in HNSC, SKCM, and COADREAD

as well as worse outcomes in ESCA and LGG highlight the importance of careful pathway and cancer type considerations when stratifying patients based on DDR-d.

To verify whether the improved survival of DDR-d cases in the MSKCC cohort was an ICI-associated effect, we examined the associations between somatic DDR mutations and survival outcomes of TCGA patients who did not receive immunotherapy, using the same Cox regression model. We identified 42 significant associations in 11 cancer types that had enough DDR-mutated cases ($n \geq 4$) for survival analysis; 28 (66.7%) of them showed worse (hazard ratio > 1) survival (Figure S5B). Somatic mutation in any of the four DDR pathways contributed to worse survival of LUSC, BRCA, COADREAD, and GBM, suggesting the positive outcomes identified in ICI-treated DDR-d patients of the same cancer types were likely specific to immunotherapy effects. When considering the 13 prioritized DDR genes altogether, mutation of DDR genes was associated with worse survival in 10 of 11 cancer types, except for BLCA (Figure S5B). Thus, the DDR-ds that we identified to be associated with improved ICI outcomes were likely predictive instead of purely prognostic.

Finally, we investigated the concordance between the DDR-associated ICI outcomes with the DDR-associated immune response signatures identified in the TCGA data. The regression coefficients of DDR mutations versus the 4 signatures, including TILs, CYT score, and *PD1*/*PD-L1* expression in TCGA, were negatively correlated with the Cox regression coefficients of DDR mutations versus ICI treatment outcome in MSKCC ($r < -0.23$, $p < 0.26$; Figures 7E–7H). These negative correlations confirmed that DDR-d tumors with high adaptive immune response corresponded with those showing improved survival upon ICI treatments, including DNA polymerase carriers in BLCA, HNSC, and STAD, as well as MMR carriers in BLCA, SKCM, and COADREAD. Notably, the tumors showing worse immunotherapy outcomes in the MSKCC ICI cohort, including damage sensor carriers in LGG and COADREAD, also showed lower levels of immune infiltrate in TCGA, suggesting potential immune evasion in these tumors that warrants further investigation (Figures 7E–7H). Overall, multiple DDR-ds were associated with both elevated tumor immune infiltrates and benefit from ICI treatment, and may be developed into biomarkers for immunotherapy.

DISCUSSION

In this study, we present a comprehensive evaluation of the immunogenic associations of germline-predisposing variants, somatic driver mutations of 80 DDR genes grouped into DDR pathways, and DNA methylations of MMR genes in 10,080 cancers (Figure 1). We also tested tumor immunogenicity in a genetically engineered ml*h1* mouse model and assessed the predictive function of DDR pathway mutations in immunotherapy-treated cancer patients (Figures 6 and 7). Germline and somatic mutations in HR genes were associated with higher TMB and neoantigen loads in *BRCA*-associated cancers, whereas somatic mutations affecting MMR, damage sensors, and DNA polymerases were associated with high tumor neoantigen hotspots and immune gene signatures across many cancer types (Figures 2, 3, and 4). Among the MMR genes, our results indicate greater importance for *MLH1* methylation to induce immunogenicity in colorectal cancer and *MSH2*/*MSH6* mutations in endometrial cancer (Figure 5). Overall, mutations in DDR genes affecting HR, damage sensors, and DNA polymerases were associated with increased immune infiltrations and higher survival after immunotherapy in BLCA, LUAD, and HNSC (Figure 7). These results suggest that different DDR pathway aberrations could elicit different extents of immune reaction in different cancer types.

HR deficiency has been shown to be associated with immunogenicity in breast cancer,[24] but the association of *BRCA1/2* mutation and immunotherapy response in non-*BRCA*-associated cancer types remains unknown.[25] Our results show that somatic mutations of *BRCA1/2* genes were associated with higher TMB, neoantigen loads, and hypermutator phenotypes even in of non-germline *BRCA*-associated cancer types, including UCEC, STAD, and COADREAD. *BRCA1/2*-deficient tumors also had higher *PD1* mRNA expression and CYT scores. Furthermore, BLCA, LUAD, HNSC, and COADREAD patients with somatic HR deficiency showed better survival after immunotherapy, suggesting the potential utility of HR deficiency as a predictive for these cancer types. However, some forms of HR deficiency in *BRCA*-associated cancer types, such as germline *BRCA1* mutation in OV and *BRCA2* mutation in BRCA, showed limited associations with TMB or neoantigen loads, and also failed to demonstrate improved survival after immunotherapy in the limited clinical cohorts available for this study.

MMR deficiency has been used as a predictive biomarker for ICI treatment; yet, a large fraction of patients with MMR alterations do not respond to immunotherapy.[13,14] We found that mutations in MMR genes showed strong gene- and cancer-specific variation in association with TMB, neoantigen load, and tumor immune response. Methylation or mutations of *MLH1* were most strongly associated with higher immune infiltrates in COADREAD and were also associated with a higher level of *PD-L1* expression and CYT score in STAD. In contrast, mutations of *MSH2* and *MSH6* showed the most significant associations with the immune infiltrates in UCEC. Most *MLH1*-deficient cancers had higher SNV-mediated and indel-mediated neoantigen loads and were associated with higher immune gene expression. These findings are consistent with recent studies using genetically engineered mouse models showing higher antitumor immune response in MMR-deficient cancers that also demonstrate high indel mutational loads.[22,26] The different levels of tumor immunogenicities associated with different MMR genes may explain some variability of treatment response across MMR-deficient tumors. Jointly considering the MMR mutation and SNV/indel neoantigen loads may better predict immunotherapy response.

Although *POLE* mutation status has been incorporated into clinical studies for ICI,[27,28] and inhibition of *ATM* and *ATR* was shown to influence immunotherapy response in model systems,[4] how DNA polymerase and damage sensor deficiencies affect the tumor immune microenvironment and sensitivity to ICI therapy is unclear. We found that mutations in genes of DNA damage sensor (*ATM*, *ATR*, and *CHEK2*) and DNA polymerase (*POLE* and *POLQ*) pathways could increase neoantigen load and MSI, and are associated with greater immune infiltration. Somatic

CellPress
OPEN ACCESS

mutations of DNA polymerase were associated with better clinical outcomes of patients who received ICI treatment. Notably, the associations of DNA damage sensor mutations and ICI treatment outcomes differed across cancer types, and associations with decreased immune infiltrates and worse ICI treatment outcomes were observed for COADREAD and LGG. DDR genes including *ATM*, *ATR*, *CHEK2*, *POLE*, and *POLQ* could serve as potential biomarkers of ICI response, but their distinct and cancer-specific effects need to be investigated further.

Overall, our analyses characterized the influence of multiple DDR-ds on genome damage, tumor immune infiltrates, and ICI treatment outcomes. The results provide candidate response biomarkers that can inform the rational design of ICI trials across multiple cancer types, potentially leading to improved immunotherapy options for DDR-d cancer cases.

Limitations of study

Several topics relevant to the immunogenicity of DDR-ds, for example MMEJ, were not included in the scope of this study. Statistical powers were limited for rare DDR-ds in our cohorts, and the variable sample sizes of different cancer types result in variable power to detect associations. In addition, our analyses focus on hypotheses centered on adaptive immunity triggered by DDR-d-associated TMB and neoantigens. However, we recognize that DDR mutations may also affect other processes not examined here, such as activation of cyclic GMP-AMP synthase-stimulator of interferon genes (cGAS-STING) signaling that also can lead to increased immune activation, even in the absence of high neoantigen expression.[29,30] Finally, observations based on human cohorts represent correlations, and the causality between DDR-ds and tumor immune response requires mechanistic investigations.

STAR★METHODS

Detailed methods are provided in the online version of this paper and include the following:

- KEY RESOURCES TABLE
- RESOURCE AVAILABILITY
  - Lead contact
  - Materials availability
  - Data and code availability
- EXPERIMENTAL MODEL AND SUBJECT DETAILS
  - Cohort description and data compilation
  - Animal experiments
- METHOD DETAILS
  - Association analyses of DDR mutations and genome damage/immune signatures
  - Independent and joint contribution of germline and somatic mutations
  - Neoantigen load distribution and neoantigen hotspots enrichment in DDR-d cases
  - Comparison of DDR-d versus immune signature association and DDR-d versus neoantigen load association
  - Hypermutators and microsatellite instability analysis
  - Determination of the mutational and epigenetic contribution to neoantigen and immune response
  - Immunotherapy clinical outcome analysis
  - Vector design
  - Hydrodynamic tail vein injection
- QUANTIFICATION AND STATISTICAL ANALYSIS
  - Multivariate regression analysis
  - Multiple comparison adjustment

SUPPLEMENTAL INFORMATION

Supplemental information can be found online at https://doi.org/10.1016/j.xcrm.2021.100276.

ACKNOWLEDGMENTS

The authors wish to acknowledge the participating patients and family who generously contributed to the datasets. The work is supported by NIH NCI R37CA230636 and NIGMS R35GM138113 from the Icahn School of Medicine at Mount Sinai, the Breast Cancer Research Foundation (Investigator award BCRF 19-133), and the Susan G. Komen Foundation (grant no. SAC160076) from Yale Cancer Center.

AUTHOR CONTRIBUTIONS

K.-l.H. and T.Q. conceived the research and designed the analyses. T.Q. conducted the TCGA analyses and T.J. conducted the patient-cohort survival analyses. M.M. helped with the statistical method. K.E.L. and A.L. conducted the *in vivo* experiments. L.P. and K.-l.H. supervised the study. T.Q., K.-l.H., and L.P. wrote the manuscript. All of the authors read, edited, and approved the manuscript.

DECLARATION OF INTERESTS

L.P. has received consulting fees and honoraria from Pfizer, AstraZeneca, Merck, Novartis, Bristol-Myers Squibb, Genentech, Eisai, Pieris, Immunomedics, Seattle Genetics, Clovis, Syndax, H3Bio, and Daiichi. A.L. has received grant support from Pfizer and Genentech for unrelated projects. The rest of the authors declare no competing interests.

Received: September 1, 2020
Revised: January 26, 2021
Accepted: April 14, 2021
Published: May 18, 2021

REFERENCES

1. Roos, W.P., Thomas, A.D., and Kaina, B. (2016). DNA damage and the balance between survival and death in cancer biology. Nat. Rev. Cancer *16*, 20–33.
2. Teo, M.Y., Bambury, R.M., Zabor, E.C., Jordan, E., Al-Ahmadie, H., Boyd, M.E., Bouvier, N., Mullane, S.A., Cha, E.K., Roper, N., et al. (2017). DNA Damage Response and Repair Gene Alterations Are Associated with Improved Survival in Patients with Platinum-Treated Advanced Urothelial Carcinoma. Clin. Cancer Res. *23*, 3610–3618.
3. Ricciuti, B., Recondo, G., Spurr, L.F., Li, Y.Y., Lamberti, G., Venkatraman, D., Umeton, R., Cherniack, A.D., Nishino, M., Sholl, L.M., et al. (2020). Impact of DNA Damage Response and Repair (DDR) Gene Mutations on Efficacy of PD-(L)1 Immune Checkpoint Inhibition in Non-Small Cell Lung Cancer. Clin. Cancer Res. *26*, 4135–4142.
4. Mouw, K.W., Goldberg, M.S., Konstantinopoulos, P.A., and D'Andrea, A.D. (2017). DNA Damage and Repair Biomarkers of Immunotherapy Response. Cancer Discov. *7*, 675–693.
5. Marcus, L., Lemery, S.J., Keegan, P., and Pazdur, R. (2019). FDA Approval Summary: Pembrolizumab for the Treatment of Microsatellite Instability-High Solid Tumors. Clin. Cancer Res. *25*, 3753–3758.

6. van Gool, I.C., Eggink, F.A., Freeman-Mills, L., Stelloo, E., Marchi, E., de Bruyn, M., Palles, C., Nout, R.A., de Kroon, C.D., Osse, E.M., et al. (2015). POLE Proofreading Mutations Elicit an Antitumor Immune Response in Endometrial Cancer. Clin. Cancer Res. *21*, 3347–3355.

7. Howitt, B.E., Shukla, S.A., Sholl, L.M., Ritterhouse, L.L., Watkins, J.C., Rodig, S., Stover, E., Strickland, K.C., D'Andrea, A.D., Wu, C.J., et al. (2015). Association of Polymerase e-Mutated and Microsatellite-Instable Endometrial Cancers With Neoantigen Load, Number of Tumor-Infiltrating Lymphocytes, and Expression of PD-1 and PD-L1. JAMA Oncol. *1*, 1319–1323.

8. Teo, M.Y., Seier, K., Ostrovnaya, I., Regazzi, A.M., Kania, B.E., Moran, M.M., Cipolla, C.K., Bluth, M.J., Chaim, J., Al-Ahmadie, H., et al. (2018). Alterations in DNA Damage Response and Repair Genes as Potential Marker of Clinical Benefit From PD-1/PD-L1 Blockade in Advanced Urothelial Cancers. J. Clin. Oncol. *36*, 1685–1694.

9. Rahman, N. (2014). Realizing the promise of cancer predisposition genes. Nature *505*, 302–308.

10. Huang, K.L., Mashl, R.J., Wu, Y., Ritter, D.I., Wang, J., Oh, C., Paczkowska, M., Reynolds, S., Wyczalkowski, M.A., Oak, N., et al. (2018). Pathogenic Germline Variants in 10,389 Adult Cancers. Cell *173*, 355–370.e14.

11. Knijnenburg, T.A., Wang, L., Zimmermann, M.T., Chambwe, N., Gao, G.F., Cherniack, A.D., Fan, H., Shen, H., Way, G.P., Greene, C.S., et al. (2018). Genomic and Molecular Landscape of DNA Damage Repair Deficiency across The Cancer Genome Atlas. Cell Rep. *23*, 239–254.e6.

12. Samstein, R.M., Lee, C.H., Shoushtari, A.N., Hellmann, M.D., Shen, R., Janjigian, Y.Y., Barron, D.A., Zehir, A., Jordan, E.J., Omuro, A., et al. (2019). Tumor mutational load predicts survival after immunotherapy across multiple cancer types. Nat. Genet. *51*, 202–206.

13. Le, D.T., Uram, J.N., Wang, H., Bartlett, B.R., Kemberling, H., Eyring, A.D., Skora, A.D., Luber, B.S., Azad, N.S., Laheru, D., et al. (2015). PD-1 Blockade in Tumors with Mismatch-Repair Deficiency. N. Engl. J. Med. *372*, 2509–2520.

14. Le, D.T., Durham, J.N., Smith, K.N., Wang, H., Bartlett, B.R., Aulakh, L.K., Lu, S., Kemberling, H., Wilt, C., Luber, B.S., et al. (2017). Mismatch repair deficiency predicts response of solid tumors to PD-1 blockade. Science *357*, 409–413.

15. Bailey, M.H., Tokheim, C., Porta-Pardo, E., Sengupta, S., Bertrand, D., Weerasinghe, A., Colaprico, A., Wendl, M.C., Kim, J., Reardon, B., et al.; MC3 Working Group; Cancer Genome Atlas Research Network (2018). Comprehensive Characterization of Cancer Driver Genes and Mutations. Cell *174*, 1034–1035.

16. Thorsson, V., Gibbs, D.L., Brown, S.D., Wolf, D., Bortone, D.S., Ou Yang, T.H., Porta-Pardo, E., Gao, G.F., Plaisier, C.L., Eddy, J.A., et al. (2018). The Immune Landscape of Cancer. Immunity *48*, 812–830, e814.

17. Roudko, V., Cimen Bozkus, C., Orfanelli, T., Blank, S.V., Greenbaum, B., and Bhardwaj, N. (2019). Widespread immunogenic poly-epitope frameshift mutations in microsatellite unstable tumors. bioRxiv. https://doi.org/10.1101/662262.

18. Jun, T., Qing, T., Dong, G., Signaevski, M., Hopkins, J., Frampton, G., Albacker, L., Cordon-Cardo, C., Samstein, R., Pusztai, L., et al. (2020). Cancer-specific associations of driver genes with immunotherapy outcome. bioRxiv. https://doi.org/10.1101/2020.06.16.155895.

19. Rooney, M.S., Shukla, S.A., Wu, C.J., Getz, G., and Hacohen, N. (2015). Molecular and genetic properties of tumors associated with local immune cytolytic activity. Cell *160*, 48–61.

20. Cortes-Ciriano, I., Lee, S., Park, W.Y., Kim, T.M., and Park, P.J. (2017). A molecular portrait of microsatellite instability across multiple cancers. Nat. Commun. *8*, 15180.

21. Niu, B., Ye, K., Zhang, Q., Lu, C., Xie, M., McLellan, M.D., Wendl, M.C., and Ding, L. (2014). MSIsensor: microsatellite instability detection using paired tumor-normal sequence data. Bioinformatics *30*, 1015–1016.

22. Germano, G., Lamba, S., Rospo, G., Barault, L., Magrì, A., Maione, F., Russo, M., Crisafulli, G., Bartolini, A., Lerda, G., et al. (2017). Inactivation of DNA repair triggers neoantigen generation and impairs tumour growth. Nature *552*, 116–120.

23. Ruiz de Galarreta, M., Bresnahan, E., Molina-Sánchez, P., Lindblad, K.E., Maier, B., Sia, D., Puigvehi, M., Miguela, V., Casanova-Acebes, M., Dhainaut, M., et al. (2019). β-Catenin Activation Promotes Immune Escape and Resistance to Anti-PD-1 Therapy in Hepatocellular Carcinoma. Cancer Discov. *9*, 1124–1141.

24. Kraya, A.A., Maxwell, K.N., Wubbenhorst, B., Wenz, B.M., Pluta, J., Rech, A.J., Dorfman, L.M., Lunceford, N., Barrett, A., Mitra, N., et al. (2019). Genomic Signatures Predict the Immunogenicity of BRCA-Deficient Breast Cancer. Clin. Cancer Res. *25*, 4363–4374.

25. Jonsson, P., Bandlamudi, C., Cheng, M.L., Srinivasan, P., Chavan, S.S., Friedman, N.D., Rosen, E.Y., Richards, A.L., Bouvier, N., Selcuklu, S.D., et al. (2019). Tumour lineage shapes BRCA-mediated phenotypes. Nature *571*, 576–579.

26. Mandal, R., Samstein, R.M., Lee, K.W., Havel, J.J., Wang, H., Krishna, C., Sabio, E.Y., Makarov, V., Kuo, F., Blecua, P., et al. (2019). Genetic diversity of tumors with mismatch repair deficiency influences anti-PD-1 immunotherapy response. Science *364*, 485–491.

27. Mehnert, J.M., Panda, A., Zhong, H., Hirshfield, K., Damare, S., Lane, K., Sokol, L., Stein, M.N., Rodriguez-Rodriquez, L., Kaufman, H.L., et al. (2016). Immune activation and response to pembrolizumab in POLE-mutant endometrial cancer. J. Clin. Invest. *126*, 2334–2340.

28. Johanns, T.M., Miller, C.A., Dorward, I.G., Tsien, C., Chang, E., Perry, A., Uppaluri, R., Ferguson, C., Schmidt, R.E., Dahiya, S., et al. (2016). Immunogenomics of Hypermutated Glioblastoma: A Patient with Germline POLE Deficiency Treated with Checkpoint Blockade Immunotherapy. Cancer Discov. *6*, 1230–1236.

29. Mackenzie, K.J., Carroll, P., Martin, C.A., Murina, O., Fluteau, A., Simpson, D.J., Olova, N., Sutcliffe, H., Rainger, J.K., Leitch, A., et al. (2017). cGAS surveillance of micronuclei links genome instability to innate immunity. Nature *548*, 461–465.

30. Chatzinikolaou, G., Karakasilioti, I., and Garinis, G.A. (2014). DNA damage and innate immunity: links and trade-offs. Trends Immunol. *35*, 429–435.

31. Ellrott, K., Bailey, M.H., Saksena, G., Covington, K.R., Kandoth, C., Stewart, C., Hess, J., Ma, S., Chiotti, K.E., McLellan, M., et al. (2018). Scalable Open Science Approach for Mutation Calling of Tumor Exomes Using Multiple Genomic Pipelines. Cell Syst. *6*, 271–281.e7.

32. Carrot-Zhang, J., Chambwe, N., Damrauer, J.S., Knijnenburg, T.A., Robertson, A.G., Yau, C., Zhou, W., Berger, A.C., Huang, K.L., Newberg, J.Y., et al. (2020). Comprehensive Analysis of Genetic Ancestry and Its Molecular Correlates in Cancer. Cancer Cell *37*, 639–654.e6.

33. Weinstein, J.N., Collisson, E.A., Mills, G.B., Shaw, K.R., Ozenberger, B.A., Ellrott, K., Shmulevich, I., Sander, C., and Stuart, J.M.; Cancer Genome Atlas Research Network (2013). The Cancer Genome Atlas Pan-Cancer analysis project. Nat. Genet. *45*, 1113–1120.

34. Ding, L., Bailey, M.H., Porta-Pardo, E., Thorsson, V., Colaprico, A., Bertrand, D., Gibbs, D.L., Weerasinghe, A., Huang, K.L., Tokheim, C., et al.; Cancer Genome Atlas Research Network (2018). Perspective on Oncogenic Processes at the End of the Beginning of Cancer Genomics. Cell *173*, 305–320.e10.

35. R Development Core Team (2009). A language and environment for statistical computing (R Foundation for Statistical Computing).

36. Sanchez, G., Trinchera, L., and Russolillo, G. (2015). plspm: Tools for Partial Least Squares Path Modeling (PLS-PM). https://rdrr.io/cran/plspm/.

37. Kassambara, A., Kosinski, M., Biecek, P., and Scheipl, F. (2021). survminer: Drawing Survival Curves. https://rpkgs.datanovia.com/survminer/#ggsurvplot-drawing-survival-curves.

38. Cong, L., Ran, F.A., Cox, D., Lin, S., Barretto, R., Habib, N., Hsu, P.D., Wu, X., Jiang, W., Marraffini, L.A., and Zhang, F. (2013). Multiplex genome engineering using CRISPR/Cas systems. Science *339*, 819–823.

CellPress
OPEN ACCESS

STAR★METHODS

KEY RESOURCES TABLE

REAGENT or RESOURCE	SOURCE	IDENTIFIER
**Bacterial and virus strains**		
Stbl3	Invitrogen	C737303
**Critical commercial assays**		
QIAquick Gel Extraction Kit	QIAGEN	Cat #28706
QIAprep Spin Miniprep Kit	QIAGEN	Cat #27106
EndoFree Plasmid Maxi Kits	QIAGEN	Cat #12362
**Deposited data**		
TCGA germline variants	Huang et al.[10]	https://gdc.cancer.gov/about-data/publications/PanCanAtlas-Germline-AWG
TCGA somatic mutations	Ellrott et al.[31]	https://gdc.cancer.gov/about-data/publications/mc3-2017
TCGA somatic mutation functional prediction	Bailey et al.[15]	https://gdc.cancer.gov/about-data/publications/pancan-driver
Genetic principal components of TCGA samples	Carrot-Zhang et al.[32]	Principle Component Analysis – WashU: https://gdc.cancer.gov/about-data/publications/CCG-AIM-2020
TCGA mRNA Expression	The Cancer Genome Atlas Research Network[33]	https://gdc.cancer.gov/about-data/publications/pancanatlas
TCGA Genomic and immune signatures	Thorsson et al.[16]	Download from Supplemental information
TCGA MSISensor score	Li et al.[34]	Download from Supplemental information
TCGA DNA damage repair genes and methylation	Knijnenburg et al.[11]	Download from Supplemental information
TCGA Hypermutators	Bailey et al.[15]	https://gdc.cancer.gov/about-data/publications/pancan-driver
Immunotherapy-treated cohort somatic and clinical	Samstein et al.[12]	http://www.cbioportal.org/study/summary?id=tmb_mskcc_2018
**Experimental models: organisms/strains**		
C57BL/6 mice, female, wild-type	Envigo	N/A
**Oligonucleotides**		
Mlh1 sgRNAs: CACCGTCACCGTGATCAGGGTGCCC,	This paper	N/A
Mlh1 sgRNAs: CACCGCAACCAGGGCACCCTGATCA	This paper	N/A
Mlh1 sgRNAs: CACCGATTGGCAAGCATAAGCCATG	This paper	N/A
**Recombinant DNA**		
pT3-EF1a-MYC-IRES-luciferase	Ruiz de Galarreta et al.[23]	N/A
pT3-EF1a-MYC-IRES-luciferase-OS	Ruiz de Galarreta et al.[23]	N/A
px330-tandem-sg-p53	This paper	N/A
px330-sg-p53	Ruiz de Galarreta et al.[23]	N/A
**Software and algorithms**		
R-project	R-project[35]	https://www.r-project.org/
plspm R package	Sanchez et al.[36]	https://github.com/gastonstat/plspm
survminer R package	Kassambara et al.[37]	https://cran.r-project.org/web/packages/survminer/index.html
In-house scripts	This paper	https://github.com/tao-qing/DDRImmune

RESOURCE AVAILABILITY

Lead contact

Further information and requests for resources and reagents should be directed to and will be fulfilled by the Lead contact, Dr. Kuan-lin Huang (kuan-lin.huang@mssm.edu).

Materials availability

All unique materials and reagents generated in this study are available from the Lead Contact with a completed material transfer agreement.

Data and code availability

The TCGA germline variants are available at https://gdc.cancer.gov/about-data/publications/PanCanAtlas-Germline-AWG. The TCGA somatic mutations are available at https://gdc.cancer.gov/about-data/publications/mc3-2017. The immune signatures and neoantigen data are available at https://gdc.cancer.gov/about-data/publications/panimmune. The in-house R scripts for regression and PLSPM analysis are available at https://github.com/tao-qing/DDRImmune. Data supporting the findings of this study are available in the Article, Supplemental information, or from the authors upon reasonable request.

EXPERIMENTAL MODEL AND SUBJECT DETAILS

Cohort description and data compilation

TCGA germline predisposing variants

We obtained 853 germlines pathogenic/likely-predisposing variants of 10,389 TCGA cancer cases, as described by Huang et al.[10]

Somatic mutations

Somatic mutations of 10,295 cases were obtained from the PanCanAtlas Multi-Center Mutation Calling in Multiple Cancers (MC3) dataset.[31] The tumor mutation burden (TMB) was calculated as the mutation counts of all the somatic mutations divided by the total length of the coding regions (https://api.gdc.cancer.gov/data/b1e303a5-a542-4389-8ddb-1d151218be75) for each TCGA individual. The somatic mutations of 299 cancer driver genes and the functional prediction information were obtained from the TCGA PanCanAtlas driver project.[15] The file "Mutation.CTAT.3D.Scores.txt" included mutation prediction score could be accessed through https://gdc.cancer.gov/about-data/publications/pancan-driver. We only considered the nonsynonymous mutations in 299 cancer driver genes, including missense, non-sense, frameshifting, in-frame shifting, or splice-site altering single-nucleotide changes or indels. Mutations predicted as functional impact by at least one algorithm described in Bailey et al.[15] or classified as truncations were considered as somatic driver mutations. We collected 35,815 likely somatic driver mutations for analyses.

Genetic principal components of TCGA cohort

We obtain the principal components (PCs) calculated by the WashU analysis in the TCGA PanCanAtlas project (https://gdc.cancer.gov/about-data/publications/CCG-AIM-2020).[10,32] The downloaded PC data were calculated based on 298,004 common variants (MAF > 0.15) with low missingness; PC1 and PC2 accounted for 51.6% and 29.2% of the variations across the first 20 PCs,[32] and were included as covariates in the regression analysis.

DNA damage repair genes and methylation data

The 80 DNA damage repair genes and DNA methylation data indicating their methylation of promoter regions (upstream and downstream 1500bp flanking regions of Transcription Start Sites (TSSs) of all annotated transcripts by UCSC) in TCGA were obtained from Knijnenburg et al.[11]

Expression data

The batched-normalized mRNA gene-expression data of TCGA samples were obtained from the PanCancer Atlas consortium, available at the publication page (https://gdc.cancer.gov/about-data/publications/pancanatlas).

Genomic and immune signatures

We integrated genomic and immune signatures of 10,260 TCGA individuals from Thorsson et al.,[16] including SNV and Indel neoantigens value, Lymphocyte Infiltration Signature (tumor-infiltrating lymphocytes, TILs). The cytolytic activity (CYT) score was calculated based on the average mRNA expression of *GZMA* and *PRF1*. The MSISensor scores reflected the status of microsatellite instability status was obtained from Ding et al.[34]

Hypermutators

We obtained 344 TCGA hypermutated samples from TCGA PanCanAtlas driver project.[15] Based on Bailey et al.,[15] the hypermutators were defined as samples with a mutation count greater than 1.5 times the interquartile range above the third quartile in their respective cancer types, and the number of mutations in a sample exceeds 1,000.

In our analysis, we only consider 10,080 TCGA cases included in both germline and somatic mutation calls that have clinical information, immune signatures, and mRNA expression data. Those samples include 9,738 non-hypermutated and 342 hypermutated cancer cases. When assessing the DDR mutation frequencies in non-hypermutators, all the germline variants and 99.9% somatic

mutations are heterozygous. Among those DDR-deficient tumors, 1.7% and 21.5% of cases carried germline and somatic mutation in multiple DDR genes, respectively. About 9.8% of DDR-deficient cases carried multiple somatic mutations in one DDR genes while DDR genes only have single germline variants.

Immunotherapy-treated cohort

Clinical and genomic data were download from Samstein et al.,[12] which included 1,661 patients who had received at least one dose of an ICI (targeting PD-1, PD-L1 or CTLA-4). Somatic exonic mutations were identified by the MSK-IMPACT panel, including 468 cancer genes. Cancer type with less than 20 patients in the cohort (e.g., uveal melanoma, chromophobe kidney cancer, papillary kidney cancer) were excluded. After filtering, 1,525 patients who had somatic mutation calls and clinical outcomes remained. The three most common cancer types in the discovery cohort were melanoma (SKCM, n = 300), lung adenocarcinoma (LUAD, n = 297), and bladder cancer (BLCA, n = 215).

Animal experiments

The 6-8 week old, wild-type, female, C57BL/6 mice were purchased from Envigo and used for all experiments. All murine experiments were approved by the Icahn School of Medicine at Mount Sinai (ISMMS) Animal Care and Use Committee (protocol no. 2014-0229). Mice were kept within specific pathogen-free conditions with food and water provided as needed. All mice were examined before experiments to verify health and acclimation.

METHOD DETAILS

Association analyses of DDR mutations and genome damage/immune signatures

We perform multivariate linear regression analysis (Equation 1) by comparing gene-level germline and somatic alterations with the neoantigens and TMB, respectively. Mutational status of DDR genes in each individual were transformed into binary matrix. We assign 1 and 0 to case with and without mutation of a gene, respectively. The neoantigens and TMB were transformed to a log2 scale and were considered as dependent variable.

We identified 13-prioritized DDR genes that showed a strong positive correlation (coefficient > 1.5, FDR < 0.05) with TMB and SNV/indel neoantigen loads. We classified those genes into four functional group including *BRCA1, BRCA2, PALB2* (HR), *MLH1, MSH2, MSH3, MSH6, PMS2* (MMR), *ATM, ATR, CHEK2* (Damage Sensor), and *POLE, POLQ* (DNA Polymerase) and further used in subsequent analyses.

Independent and joint contribution of germline and somatic mutations

We tailored a PLS-PM analysis to investigate the independent and joint contribution of germline variants and somatic mutations. The PLS-PM is a multivariate data analysis method which introduces latent variables for analyzing systems of relationships between multiple variables. Thirteen genes (*BRCA1, BRCA2, PALB2, MLH1, MSH2, MSH3, MSH6, PMS2, ATM, ATR, CHEK2, POLE,* and *POLQ*) were included in the PLS-PM model. We introduce two latent variables (germline and somatic), which indicates the combined effect of germline variants and somatic mutations. The path coefficients of individual genes were estimated by ordinary least-squares in the multiple regression. The coefficient of the latent variable was calculated by the ordinary least-squares type algorithm. We only considered cancer types with at least four individuals carried germline variants or somatic mutations. For TMB and neoantigen analysis, we only performed PLS-PM analysis with 9,738 non-hypermutated cases. The PLS-PM analysis was performed with the R package "plspm."[36]

Neoantigen load distribution and neoantigen hotspots enrichment in DDR-d cases

We grouped DDR genes by their pathway functions, including MMR, HR, Damage Sensors, and DNA Polymerase. Non-hypermutated cases were assigned to mutated and wild-type if carried germline or somatic mutations. To avoid overlap, cases were excluded if they carried mutations of more than two pathways. We compare the distribution of log2 transformed SNV/Indel neoantigen load in germline (g) and somatic (s) affected cases in each DDR-pathway group versus the wild-type cases using the two-side Wilcox rank-sum tests.

Given the low prevalence of individual neoantigens in human cancer, we analyzed neoantigen hotspots across all cancer types. The SNV and indel neoantigen hotspots were defined as neoantigen identified in $\geq$ 5% of 9,738 non-hypermutated cases. We estimated the enrichment of neoantigen hotspots in cases with DDR mutation versus that of DDR wild-type cases using two-sided Fisher's exact tests.

Comparison of DDR-d versus immune signature association and DDR-d versus neoantigen load association

The association coefficients of DDR-d versus immune signature and DDR-d versus neoantigen load were estimated within each cancer type using multivariate linear regression in Equation 1 for each DDR pathway, including MMR, HR, Damage Sensors, and DNA Polymerase. Then, we compared the two linear regression coefficient using Pearson's correlation analysis. The Pearson's correlation coefficient (r) was used to measure of the strength of the association and the p value was estimated based on the Pearson product-moment correlation coefficient.

Hypermutators and microsatellite instability analysis

We investigate the enrichment of DDR alteration in hypermutators and their contribution to MSI in all 10,080 cases, including 342 hypermutators. We construct two-way contingency tables for gene status (1 and 0 represent mutant and wild-type, respectively) and hypermutated status (1 means hypermutator, 0 means non-hypermutator). The two-sided Fisher's exact test was used to evaluate the enrichment of germline variant and somatic mutation of the 13-prioritized DDR genes. MSIsensor score is estimated based on the exome sequencing data, which represents the microsatellite instability in tumor tissues.[21] The MSIsensor was transformed to a log2 scale, and to avoid infinite values, a value of 0.01 was added to the MSIsensor value of each sample before log2 transformation. We compared DDR mutated (MUT, with at least one germline variant or somatic mutations in any of 13 DDR genes) and wild-type (WT, without DDR alteration) cases using Wilcoxon rank-sum test for each TCGA cancer type. PLS-PM analysis was performed with the 13 DDR genes to estimate the germline and somatic contribution to MSI.

Determination of the mutational and epigenetic contribution to neoantigen and immune response

We evaluated germline variants, somatic mutations, and DNA methylations for six MMR genes (*MLH1, MSH3, MSH2, MSH6,* and *PMS2*) in 32 TCGA cancer types. Given that germline variants rarely affect MMR genes, we merged germline variants with somatic mutations annotated as "mut," whereas "me" represented DNA methylation. We compared the neoantigen loads between cases harbored any of the genomic alterations in an MMR gene with those without MMR alterations using two-sided Wilcoxon rank-sum tests. To evaluate the effect of methylation and mutation of individual MMR genes to tumor immune infiltrate signatures, we performed multivariate linear regression analyses of immune signatures against MMR alterations and covariates in COADREAD, STAD, UCEC (Equation 2).

Immunotherapy clinical outcome analysis

We evaluated the predictive role of somatic mutations of 13 DDR genes for the clinical outcome of ICI treatment. The primary predictor was the presence or absence of the specific variant (compared to those without the mutation) in individual DDR genes or DDR pathways (MMR, HR, Damage Sensor, DNA polymerase). Cox proportional hazards regression analysis was performed using the survival package in R. Covariates were age group at diagnosis, sex, ICI class (anti-*CTLA-4*, anti-*PD-1*/*PD-L1*, or a combination). Separate models were constructed for each cancer type. Kaplan-Meier plots were generated using the survminer and ggplot2 packages in R.[37]

To demonstrate that the immunogenicity of DDR-deficiency could further determine clinical outcomes of ICI treatment, we evaluate the concordance of DDR-d versus immune signature (TCGA) and DDR-d versus survival (MSKCC). We compared the TCGA coefficient of DDR-d versus immune signature with the MSKCC hazard ratio using Pearson's correlation analysis. Pearson's correlation coefficient (r) was used to measure the strength of the association, and Spearman's Rho test (two-sided) was used to generate the p value.

Vector design

The *pT3-EF1a-MYC-IRES-luciferase* and *pT3-EF1a-MYC-IRES-luciferase-OS* plasmids were generated previously.[23] The *px330-tandem-sg-p53* was generated by introducing the *U6-sg-p53* portion from *px330-sg-p53*[22,38] into the original *px330* vector opened by XbaI and KpnI.

To generate the *px330-sg-p53;sg-Mlh1* tandem CRISPR vectors, the *px330-tandem-sg-p53* plasmid was digested with BbsI (NEB, Cat #R0539S), gel-purified using QIAquick Gel Extraction Kit (QIAGEN, Cat #28706). Single guide RNA (sgRNA) oligos targeting the *Mlh1* gene were phosphorylated (T4 Polynucleotide Kinase, NEB Cat #M0201S) and annealed (T4 DNA Ligase, NEB Cat #M0202S) into the opened *px330-tandem-mp53-1* vector. Three sgRNAs were used to target the *Mlh1* gene: CACCGTCACCGTGAT CAGGGTGCCC, CACCGCAACCAGGGCACCCTGATCA, and CACCGATTGGCAAGCATAAGCCATG. Each sgRNA was individually cloned into the *px330-tandem-sg-p53* vector, resulting in a total of three *px330-sg-p53;-sg-Mlh1* tandem CRISPR vectors. Each vector was transformed into Stbl3 bacteria, colonies were chosen for QIAprep Spin Miniprep Kit (QIAGEN, Cat #27106), and sequences were confirmed (Psomagen Inc, USA). EndoFree Plasmid Maxi Kits (QIAGEN, Cat #12362) were performed on the final *px330-sg-p53;sg-Mlh1* tandem CRISPR vectors.

Hydrodynamic tail vein injection

Optimized concentrations of vectors, generally 10 or 12 μg/mouse, were prepared in sterile 0.9% NaCl solution; the SB13 transposase-encoding plasmid was included in the mix at a 4:1 ratio of the transposon-based vector. A volume corresponding to 10% of the body weight of the mouse was injected into the lateral tail vein in around 5 s. Vectors for hydrodynamic delivery were produced using Endo-Free Plasmid Maxi Kits (QIAGEN, Cat #12362). All vector constructs were verified by sequencing and restriction enzyme digestion.

QUANTIFICATION AND STATISTICAL ANALYSIS

Multivariate regression analysis

We use a linear regression model to evaluate the effect of germline variants and somatic mutations on the TMB, neoantigens and immune signatures with the "glm" function of the "base" package of the R-project.[35] We use the glm parameter "family= gaussian()"

CellPress
OPEN ACCESS

for regression analysis in R adjusting by age at diagnosis and population substructure (first two principal components of germline genetic analysis, PC1, PC2). The model is:

$$\textit{Immune signature} \sim \textit{genetic alterations}\ (0,1) + Age + PC1 + PC2 \qquad \text{(Equation 1)}$$

where immunogenic features include TMB, neoantigen loads, or immune gene expression/signatures, and genetic alterations include germline predisposing variants or somatic driver mutations in the analyzed gene. Only genes with alterations harbored at least four individuals will be included in the regression analysis for the cancer type.

We also use a linear regression model to evaluate the effect of individual MMR gene alterations on immune signatures with the "glm" function of the "base" package of the R-project.[35] The MMR gene genomic alterations include germline or somatic mutations (mut) and DNA methylations (me). We use the glm parameter "family= gaussian()" for regression analysis in R adjusting by age at diagnosis and population substructure (first two principal components of germline genetic analysis, PC1, PC2).

$$\textit{Immune signature} \sim mutMLH1 + meMLH1 + mutMSH3 + mutMSH2 + mutMSH6 + mutPMS + Age + PC1 + PC2 \qquad \text{(Equation 2)}$$

The immune signatures included TILs, *PD1*, *PD-L1,* and CYT score. Only genes with at least four individuals carried mutations or methylation in the cancer cohort were included in the model.

Multiple comparison adjustment

All FDRs were calculated using the Benjamini & Hochberg method for multiple comparisons across all the cancer types. The significant associations were defined as FDR < 0.05, respectively. All the significant values shown in figures denote the FDR. Given some association analyses have extremely small FDR, we set 1.0×10^{-50} as the minimum value of FDR for these less than 1.0×10^{-50}.

Cell Reports Medicine, Volume *2*

Supplemental information

Diverse immune response of DNA damage repair-deficient tumors

Tao Qing, Tomi Jun, Katherine E. Lindblad, Amaia Lujambio, Michal Marczyk, Lajos Pusztai, and Kuan-lin Huang

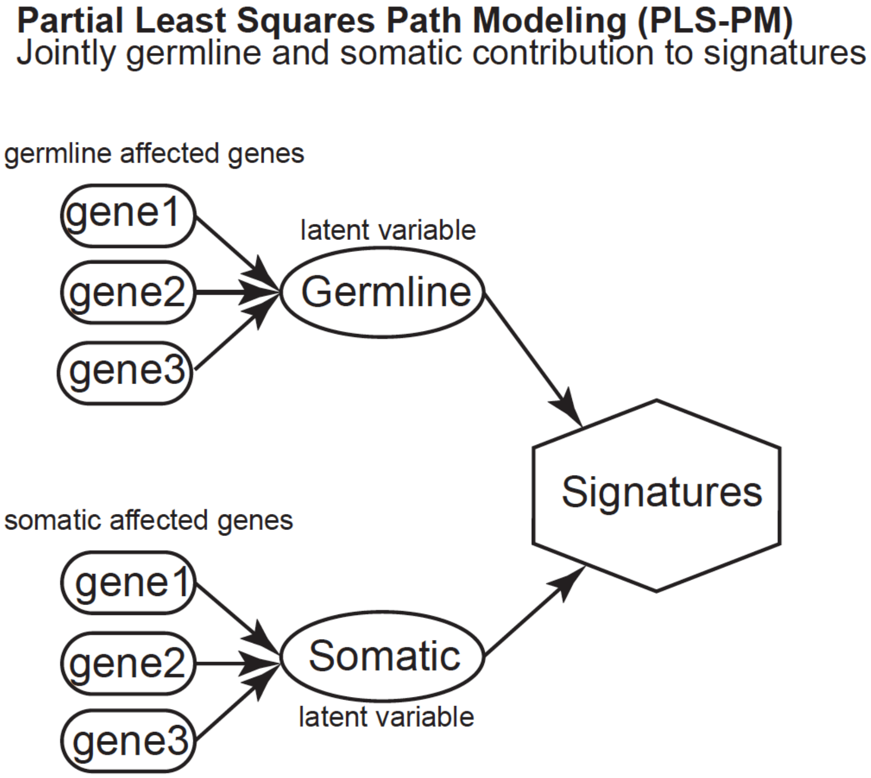

Figure S1. A schema of Partial Least Squares Path Modeling analysis, related to Figure 2E. Two latent variables are introduced, representing the combined effects of germline affected genes and somatic affected genes. The signatures indicate genome damage signatures, including tumor mutation burden, SNV, and indel neoantigen loads.

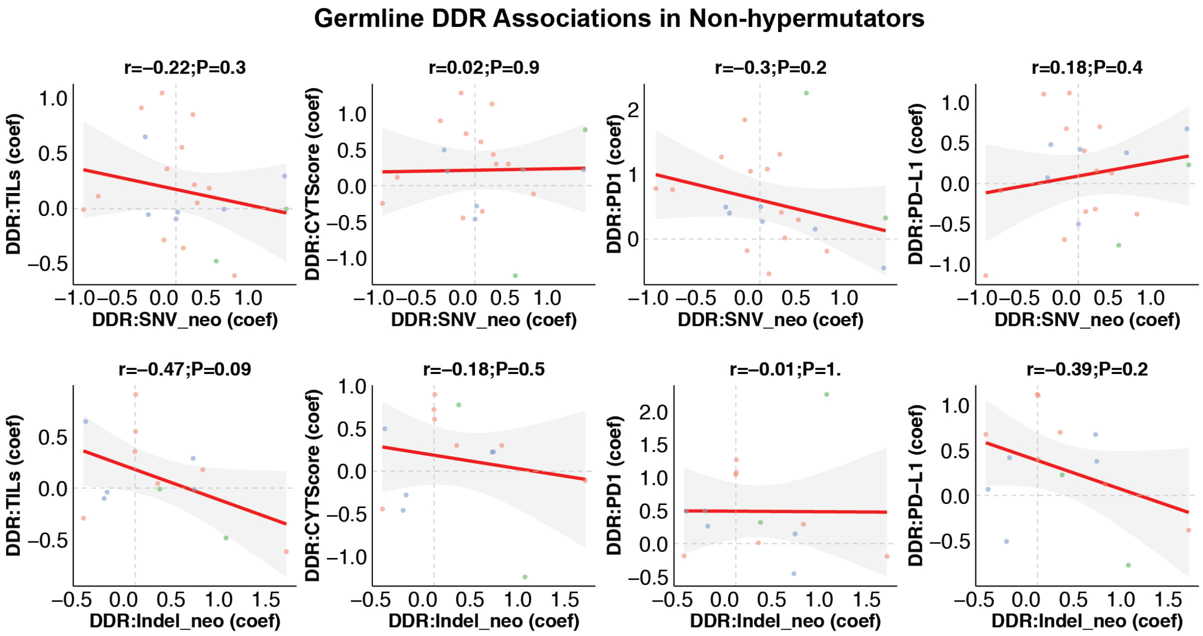

Figure S2. The association between germline DNA damage repair and tumor immune response in non-hypermutated cases, related to Figure 4. A-D. Comparisons of the correlation coefficients of germline DDR versus SNV neoantigen load with the germline DDR versus immune signature, including **A.** TILs, **B.** CYT score, **C.** *PD1* expression, and **D.** *PD-L1* expression. **E-H**. Comparisons of the correlation coefficients of germline DDR versus Indel neoantigen load with the germline DDR versus immune signature, including **E.** TILs, **F.** CYT score, **G.** *PD1* expression, and **H.** *PD-L1* expression. The *r* represents the Pearson correlation coefficient. Color of the dot represents the DDR pathways, including MMR (*MLH1*, *MSH2*, *MSH3*, *MSH6*, and *PMS2*), HR (*BRCA1/2, PALB2*), Sensor (*ATM, ATR,* and *CHEK2*) and Polymerase (*POLE* and *POLQ*). The axes indicate the correlation coefficients (coef) of linear regression adjusted by patients' age and genetic components (*immune signature/neoantigen load ~ germline DDR variants+Age+PC1+PC2*).

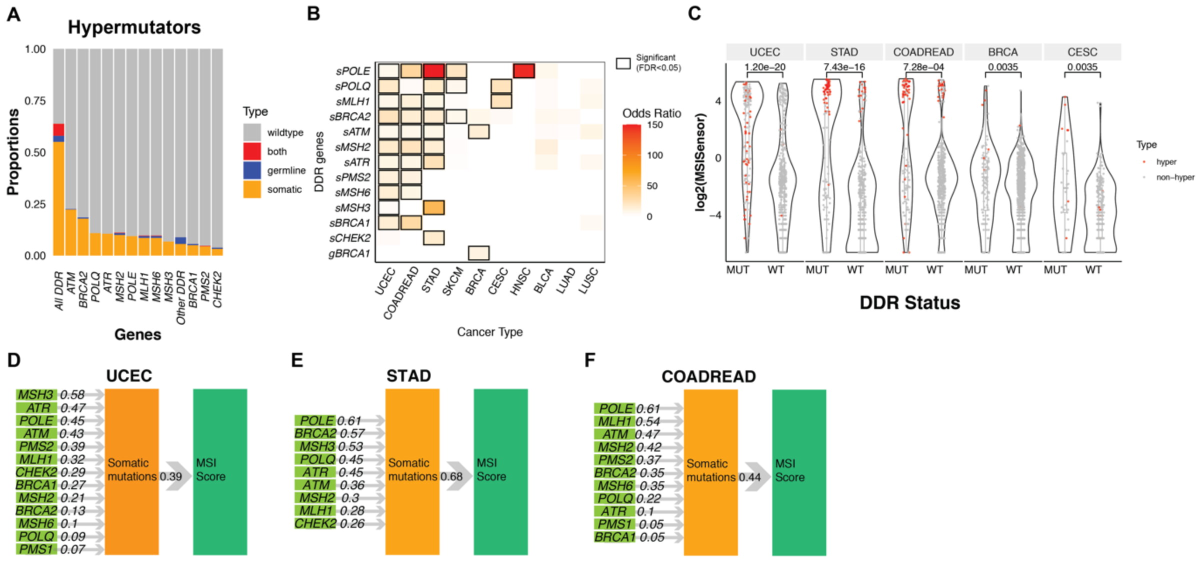

Figure S3. The effect of germline and somatic DNA damage repair mutations on hypermutator and microsatellite instability, related to Figure 2 & 3. A. The frequency of hypermutators carrying germline variants or somatic mutations in any of the 80 DDR genes. **B.** The enrichment of 13-prioritized DDR gene mutations in hypermutators, where the FDR was calculated based on results of two-side Fisher's exact tests. Only cancer types with at least one FDR<0.15 were shown. **C.** The comparison of MSISensor scores in DDR mutated and wildtype samples in each cancer type based on the Wilcox-rank-sum test. The numbers on the top of the violin plots denote FDRs resulting from the comparison. Only show cancer types with FDR<0.05 were shown. **D-F.** PLS-PM models of somatic mutations of DDR genes and MSI score (germline level was only show, because no significant association was identified in the germline level), including **D.** UCEC, **E.** STAD, **F.** COADREAD. The numbers indicated correlation coefficients identified by the PM-PLS model. Coefficients between germline variant/somatic mutation nodes and MSI nodes indicate the combined associations of the listed DDR genes.

A

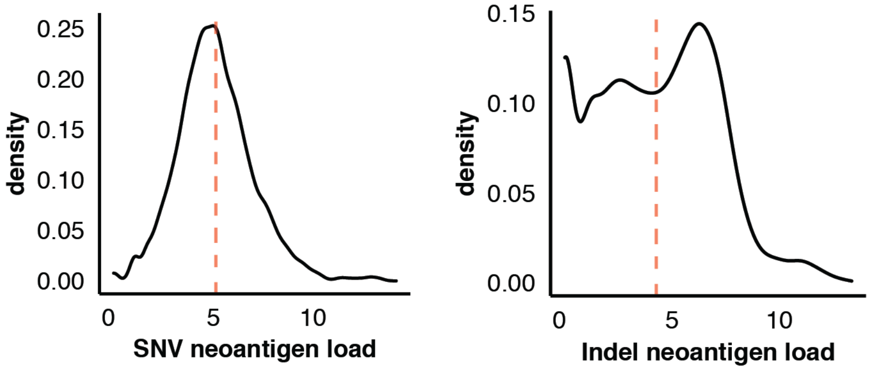

B

COADREAD

Odds Ratio=46.5 , *P*<2.2e-16

	SNV Neo High	SNV Neo Low
MLH1-deficiency	54 (11.2%)	4 (1.0%)
MLH1-wildtype	79 (16.4%)	344 (71.4%)

COADREAD

Odds Ratio=19.1 , *P*=1.46e-14

	Indel Neo High	Indel Neo Low
MLH1-deficiency	51 (11.5%)	4 (0.9%)
MLH1-wildtype	155 (35.0%)	233 (52.6%)

Figure S4. Neoantigen load and immunogenicity of *MLH1*-deficiency tumor, related to Figure 6. **A.** The distribution of log2-scale SNV and indel neoantigen load. The red line indicates the average neoantigen load for stratifying neoantigen high and low samples. **B.** contingency tables for *MLH1*-deficiency (germline variants, somatic mutations, and DNA methylation) and SNV/indel neoantigens. *P*-values were calculated by two-sided Fisher exact test.

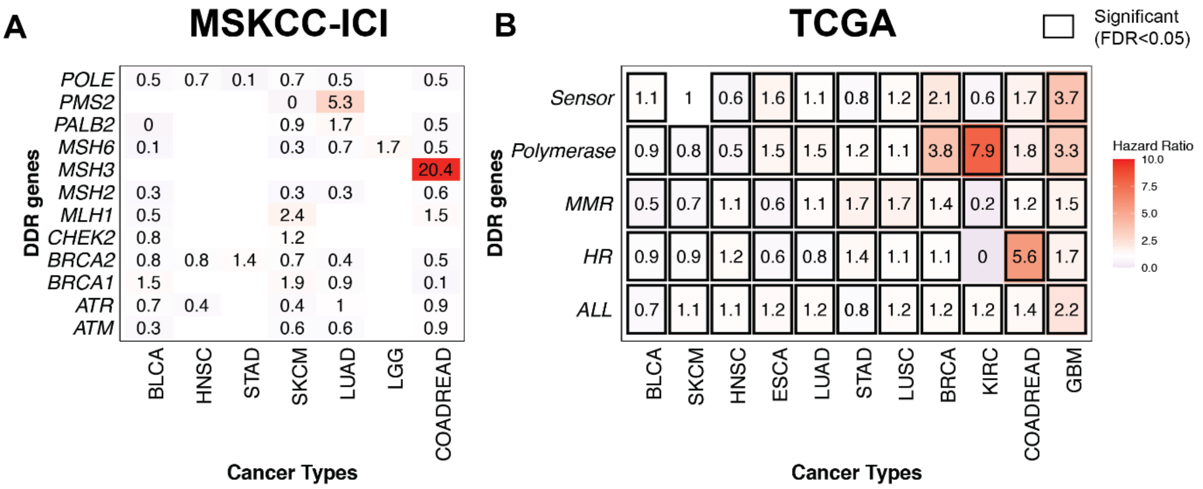

Figure S5. Associations between patients' outcome and somatic mutations of the 13-prioritized DDR genes, related to Figure 7. A. The heatmap shows the hazard ratio of individual DDR genes in the MSKCC ICI treatment cohort. The X-axis represents cancer types, and the Y-axis represents somatic mutations in each prioritized DDR gene. **B.** The heatmap shows the hazard ratio of DDR pathways in the MSKCC ICI treatment cohort. The Y-axis represents somatic mutations in DDR pathways of prioritized DDR genes, including Mismatch Repair (MMR), Homologous Recombination (HR), Damage Sensor (Sensor), DNA Polymerase (Polymerase), and all the 13-prioritized DDR genes (ALL). The value in each cell denotes the hazard ratio. The hazard ratio > 1 and < 1 suggests the association of DDR mutations with worse and better survival, respectively. Hazard ratio and *P* values were calculated using multivariable Cox proportional hazards models. Black boxes indicate FDR meet the criteria of less than 0.05 (significant).

Supplementary Table1: TCGA samples, related to Figure 1.

NO.	Cancers	Abbreviation	# All Samples	# Hypermutators
1	Glioblastoma multiforme	GBM	390	3
2	Ovarian serous cystadenocarcinoma	OV	387	1
3	Lung adenocarcinoma	LUAD	515	27
4	Lung squamous cell carcinoma	LUSC	485	16
5	Prostate adenocarcinoma	PRAD	497	2
6	Uterine Corpus Endometrial Carcinoma	UCEC	529	51
7	Bladder Urothelial Carcinoma	BLCA	411	18
8	Testicular Germ Cell Tumors	TGCT	129	0
9	Esophageal carcinoma	ESCA	184	3
10	Pancreatic adenocarcinoma	PAAD	177	1
11	Kidney renal papillary cell carcinoma	KIRP	281	0
12	Liver hepatocellular carcinoma	LIHC	363	3
13	Cervical squamous cell carcinoma and endocervical adenocarcinoma	CESC	289	14
14	Sarcoma	SARC	236	2
15	Breast invasive carcinoma	BRCA	1012	12
16	Thymoma	THYM	123	1
17	Mesothelioma	MESO	82	0
18	Colon Rectum adenocarcinoma	COADREAD	527	64
19	Stomach adenocarcinoma	STAD	439	67
20	Skin Cutaneous Melanoma	SKCM	466	44
21	Cholangiocarcinoma	CHOL	36	1
22	Kidney renal clear cell carcinoma	KIRC	368	0
23	Thyroid carcinoma	THCA	492	0
24	Head and Neck squamous cell carcinoma	HNSC	507	5
25	Brain Lower Grade Glioma	LGG	511	1
26	Kidney Chromophobe	KICH	66	1
27	Uterine Carcinosarcoma	UCS	57	2
28	Adrenocortical carcinoma	ACC	92	2
29	Pheochromocytoma and Paraganglioma	PCPG	179	0
30	Uveal Melanoma	UVM	80	0
31	Lymphoid Neoplasm Diffuse Large B-cell Lymphoma	DLBC	37	0
32	Acute Myeloid Leukemia	LAML	133	1

Supplementary Table2: core DNA damage repair genes, related to Figure 1

DNA Damage Repair Pathways	Genes	#Gene
Base Excision Repair (BER)	APEX1,APEX2,FEN1,PARP1,POLB,TDG,TDP1,UNG	8
Nucleotide Excision Repair (NER, including TC-NER and GC-NER))	CUL5,ERCC1,ERCC2,ERCC4,ERCC5,ERCC6,POLE, POLE3,XPA,XPC	10
Mismatch Repair (MMR)	EXO1,MLH1,MLH3,MSH2,MSH3,MSH6,PMS1,PMS2	8
Fanconi Anemia (FA)	FANCA,FANCB,FANCC,FANCD2,FANCI,FANCL, FANCM,UBE2T	8
Homologous Recombination (HR)	BARD1,BLM,BRCA1,BRCA2,BRIP1,EME1,GEN1,MRE11A,MUS81,NBN,PALB2,RAD50,RAD51,RAD52, RBBP8,SHFM1,SLX1A,TOP3A,TP53BP1,XRCC2, XRCC3	21
Non-homologous End Joining (NHEJ)	LIG4,NHEJ1,POLL,POLM,PRKDC,XRCC4,XRCC5, XRCC6	8
Direct Repair (DR)	ALKBH2,ALKBH3,MGMT	3
Translesion Synthesis (TLS)	POLN,POLQ,REV1,REV3L,SHPRH	5
Damage Sensor etc.	ATM,ATR,ATRIP,CHEK1,CHEK2,MDC1,RNMT, TOPBP1,TREX1	9
